# Development of neonatal-specific sequences for portable ultralow field magnetic resonance brain imaging: a prospective, single-centre, cohort study

**DOI:** 10.1016/j.eclinm.2023.102253

**Published:** 2023-10-10

**Authors:** Paul Cawley, Francesco Padormo, Daniel Cromb, Jennifer Almalbis, Massimo Marenzana, Rui Teixeira, UNITY Consortium, Sean C. Deoni, Emil Ljungberg, Carly Bennallick, Shannon Kolind, Doug Dean, Michael S. Pepper, Lydia Sekoli, Alexica De Canha, Jeanne Van Rensburg, Derek K. Jones, Niall Bourke, Hemmen Sabir, Samson Lecurieux Lafayette, Alena Uus, Jonathan O’Muircheartaigh, Steven C.R. Williams, Serena J. Counsell, Tomoki Arichi, Mary A. Rutherford, Joseph V. Hajnal, A. David Edwards

**Affiliations:** a Centre for the Developing Brain, School of Biomedical Engineering and Imaging Sciences, King’s College London, London SE1 7EH, UK; b Neonatal Intensive Care Unit, Evelina Children’s Hospital London, St Thomas’ Hospital, 6th Floor North Wing, Westminster Bridge Road, London SE1 7EH, UK; c MRC Centre for Neurodevelopmental Disorders, King’s College London, London SE1 1UL, UK; d Medical Physics, Guy’s & St. Thomas' NHS Foundation Trust, London, UK; e Hyperfine, Inc., 351 New Whitfield St., Guilford, Connecticut 06437, USA; f Centre for Neuroimaging Sciences, King’s College London, De Crespigny Park, London SE5 8AF, UK; g Department of Forensic and Neurodevelopmental Science, Institute of Psychiatry, Psychology and Neuroscience, King’s College London, London, UK; h Paediatric Neurosciences, Evelina London Children’s Hospital, Guy’s and St Thomas’ NHS Foundation Trust, London SE1 7EH, UK

**Keywords:** Low field, Portable, Magnetic resonance imaging, Neonatal, Intensive care

## Abstract

**Background:**

Magnetic Resonance (MR) imaging is key for investigation of suspected newborn brain abnormalities. Access is limited in low-resource settings and challenging in infants needing intensive care. Portable ultralow field (ULF) MRI is showing promise in bedside adult brain imaging. Use in infants and children has been limited as brain-tissue composition differences necessitate sequence modification. The aim of this study was to develop neonatal-specific ULF structural sequences and test these across a range of gestational maturities and pathologies to inform future validation studies.

**Methods:**

Prospective cohort study within a UK neonatal specialist referral centre. Infants undergoing 3T MRI were recruited for paired ULF (64mT) portable MRI by convenience sampling from the neonatal unit and post-natal ward. Key inclusion criteria: 1) Infants with risk or suspicion of brain abnormality, or 2) preterm and term infants without suspicion of major genetic, chromosomal or neurological abnormality. Exclusions: presence of contra-indication for MR scanning. ULF sequence parameters were optimised for neonatal brain-tissues by iterative and explorative design. Neuroanatomic and pathologic features were compared by unblinded review, informing optimisation of subsequent sequence generations in a step-wise manner. Main outcome: visual identification of healthy and abnormal brain tissues/structures. ULF MR spectroscopy, diffusion, susceptibility weighted imaging, arteriography, and venography require pre-clinical technical development and have not been tested.

**Findings:**

Between September 23, 2021 and October 25, 2022, 102 paired scans were acquired in 87 infants; 1.17 paired scans per infant. Median age 9 days, median postmenstrual age 40^+2^ weeks (range: 31^+3^–53^+4^). Infants had a range of intensive care requirements. No adverse events observed. Optimised ULF sequences can visualise key neuroanatomy and brain abnormalities. In finalised neonatal sequences: T2w imaging distinguished grey and white matter (7/7 infants), ventricles (7/7), pituitary tissue (5/7), corpus callosum (7/7) and optic nerves (7/7). Signal congruence was seen within the posterior limb of the internal capsule in 10/11 infants on finalised T1w scans. In addition, brain abnormalities visualised on ULF optimised sequences have similar MR signal patterns to 3T imaging, including injury secondary to infarction (6/6 infants on T2w scans), hypoxia-ischaemia (abnormal signal in basal ganglia, thalami and white matter 2/2 infants on T2w scans, cortical highlighting 1/1 infant on T1w scan), and congenital malformations: polymicrogyria 3/3, absent corpus callosum 2/2, and vermian hypoplasia 3/3 infants on T2w scans. Sequences are susceptible to motion corruption, noise, and ULF artefact. Non-identified pathologies were small or subtle.

**Interpretation:**

On unblinded review, optimised portable MR can provide sufficient contrast, signal, and resolution for neuroanatomical identification and detection of a range of clinically important abnormalities. Blinded validation studies are now warranted.

**Funding:**

The 10.13039/100000865Bill and Melinda Gates Foundation, the 10.13039/501100000265MRC, the 10.13039/100004440Wellcome/EPSRC Centre for Medical Engineering, the 10.13039/501100022431MRC Centre for Neurodevelopmental Disorders, and the 10.13039/501100000272National Institute for Health Research (NIHR) Biomedical Research Centres based at Guy’s and St Thomas’ and South London & Maudsley NHS Foundation Trusts and 10.13039/100009360King's College London.

**Research in context**

**Evidence before this study**

We undertook a wide-literature search to identify the current evidence-base for perinatal ultralow field (ULF) portable brain Magnetic Resonance Imaging (MRI). Using Embase (1978 to December 30, 2022) and Ovid MEDLINE (1948 to December 30, 2022) we searched the keyword terms “ultralow field” (OR “ultra-low field” OR “Portable” OR “Accessible”) AND “Magnetic Resonance” (OR “MRI”) AND “Infant∗” (OR “pediatric” OR “paediatric” OR “newborn∗” OR “neonat∗” OR “perinatal” OR “NICU” OR “PICU”). No previous studies have investigated optimisation of ULF sequences for structural imaging of the neonatal brain. Two papers, including one by our group, have performed relaxometry of developing neonatal brain tissues at ULF, providing quantitative data that can be used to inform structural sequence optimisations. One paediatric study has used high-field derived MRI tissue masks to replicate brain volumetry on ULF imaging, in a non-neonatal cohort. Three non-neonatal paediatric case-series have described use of low-resolution, and restricted field of view, MRI guided neurosurgery using operating-room mounted scanners (120–150 mT). One neonatal case-series has demonstrated safe use of portable ULF MR brain imaging in 14 infants, all >2 kg, whilst identifying technical challenges with need for imaging optimisation of pulse-sequences for use on neonates. Thus, hitherto ULF MRI has been principally designed to image adult brains and to answer adult clinical questions. These adult MRI sequences produce poor and inadequate results in the newborn.

**Added value of this study**

We have undertaken comprehensive optimisation of MRI sequences for the neonatal brain at ultralow field, for both T1 and T2 weighted contrasts. We show that optimised sequences are able to identify and differentiate key brain tissues and neuroanatomical structures. Additionally, advanced neonatal specific MRI sequences have been applied in a range of infant weights and gestational ages, and in the presence of common perinatal brain abnormalities with promising unblinded performance.

**Implications of all the available evidence**

ULF MRI has promise to benefit a wide range of neonatal clinical and research applications; with potential for advantage greatest within vulnerable infant populations most in need–both in high resourced intensive care settings, where transport of sick infants to remote scanning departments has attributable clinical risk, and, importantly, within low-income settings, where MRI access may be poor or non-existent. With the use of bespoke neonatal optimised pulse-sequences, this technology is ready for formal evaluation in the neonatal population globally, with blinded comparison against the established means of neuroimaging - high-quality cranial ultrasound, and reference-standard MRI.

## Introduction

Newborn infants with congenital brain anomalies and those exposed to perinatal insults such as infection, prematurity or hypoxia-ischaemia are at high risk of neurodevelopmental impairment, special educational needs and neurodisabilities.^1^, ^2^, ^3^, ^4^, ^5^, ^6^ The number of infants affected is significant: rates of preterm birth globally are estimated at 5–18%, with highest incidences in low- and middle-income countries^7^; while the incidence of neonatal encephalopathy is 8.5 cases per 1000 live births.^8^

The diagnosis and management of these conditions depend critically on neuroimaging.^9^, ^10^, ^11^ Whilst cranial ultrasound scanning (USS) is a widely available and established bedside modality that provides key information on brain anatomy, the ventricular system and detection of some pathologies, Magnetic Resonance (MR) imaging remains the gold standard for aetiological and prognostic assessment.^12^^,^^13^ Current diagnostic MR imaging typically utilises either 1.5 Tesla (T) or higher 3T static magnetic field strengths. These MR scanners have a high financial cost and require considerable infrastructure, including: liquid helium to maintain the superconductivity of the magnet, reliable-continuous high-power electricity supply, reinforced flooring, electromagnetic shielding, and a large fringe field (5 Gauss) safety exclusion radius. MR scanners are therefore usually based in radiology departments remote from nurseries or neonatal intensive care units. Both the cost and the location of current MR facilities severely limits access for vulnerable newborn infants.

In contrast, new permanent magnet designs at ultralow magnet strength (<0.1 T) facilitate manufacture at lower cost, with reduced ongoing energy and infrastructure requirements. These specialised ultralow field (ULF) systems can be portable and could increase MR accessibility and proximity; providing access to MR technology in low- and middle-income countries (LMIC) and allowing bed-side imaging within high-resource environments.^14^, ^15^, ^16^, ^17^ The ability to perform point-of-care MR imaging would be hugely advantageous to preterm infants and those receiving critical care, where transport to off-site imaging suites presents clinical risk, including potential cardiorespiratory compromise and hypothermia. At present, significant resource is required to mitigate these risks.^18^^,^^19^

ULF systems have been trialled in adult and paediatric settings.^17^^,^^20^, ^21^, ^22^, ^23^ However, imaging infants presents specific practical and clinical challenges. Furthermore, neonatal brain tissue undergoes a period of intense development with markedly different MR properties compared to mature tissue, and thus ULF MR image acquisitions designed for adults and older children do not provide sufficient contrast when imaging the neonatal brain.^20^^,^^21^^,^^23^

Furthermore, whilst reducing the static magnetic field strength facilities the practicability of portable MR systems it sacrifices signal strength—thus ultra-low field systems will have limits in what they can achieve in signal, contrast and resolution when compared with higher field strengths. The boundary or limitation of low-field capability in neonatal MR brain imaging has not been tested.

Bespoke neonatal MR sequences are needed with optimised parameters which accommodate the different MR relaxation times of the developing brain.^20^^,^^24^^,^^25^ This is crucial, adult optimised sequences produce poor and inadequate results in the newborn and further study into the diagnostic efficacy of ULF imaging should therefore not proceed until such prior technical development for neonatal use is undertaken.^26^

In this study, we aimed to: 1) use iterative explorative design to develop neonatal-specific ULF sequences optimised for brain imaging, 2) use side-by-side visual comparison with paired reference-standard high-field MR, across a range of gestational maturities and pathologies, to discover if healthy brain tissues, known neuro-anatomical changes and abnormalities are identifiable at 64mT and, if so, learn how appearances may differ at 64mT acquisition, 3) dependent on the putative performance of neonatal optimised sequences, determine if the potential of this technology would warrant future global validation studies, 4) if warranted, provide candidate sequences and base data to inform planning of such studies.

## Methods

### Study design

Prospective cohort study within a UK neonatal specialist referral centre. Newborn infants undergoing 3T MR brain imaging for risk or suspicion of brain abnormality, or for research into healthy newborn brain development, at the Evelina London Children’s Hospital Newborn Imaging Centre were recruited for paired ULF 64mT MR imaging by convenience sampling of all infants whose parents provided informed written consent. Recruitment took place between September 23, 2021 and October 25, 2022. UK Ethics approvals: 12/LO/1247—West London & GTAC Research Ethics Committee, and 19/LO/1384—City & East Research Ethics Committee.

**Fig. 1:**
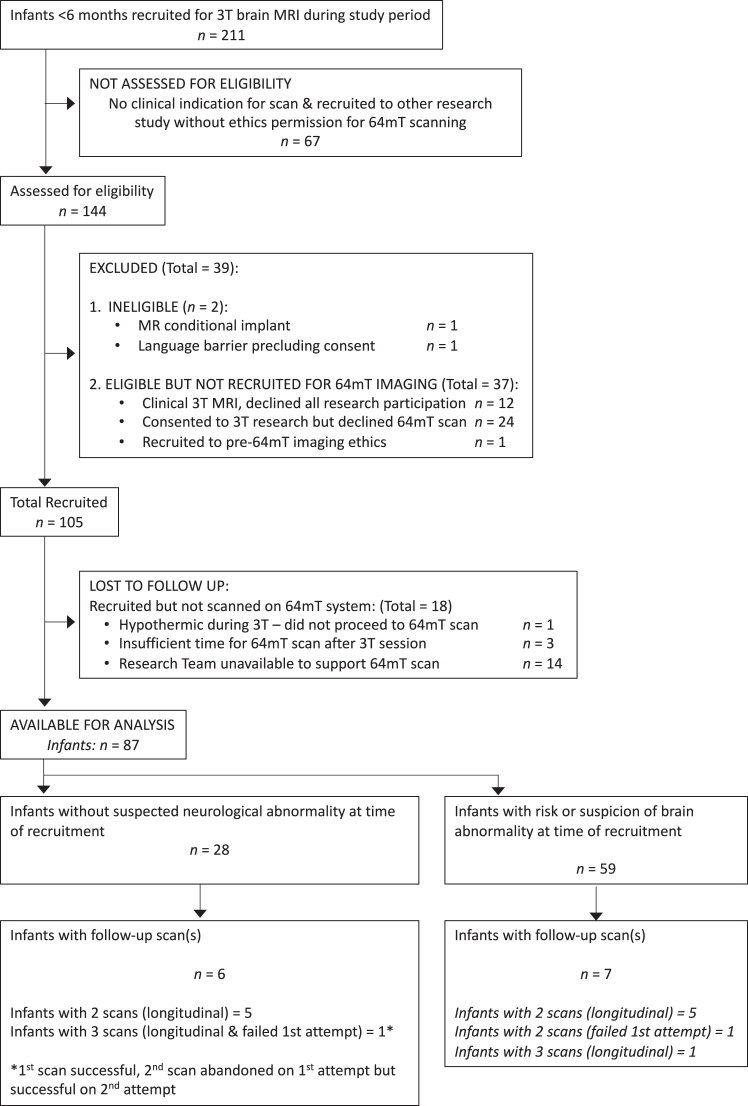
**Patient recruitment flow diagram.** 102 scans (including 2× failed attempts) in 87 participants.

The STROBE^27^ statement was used to aid reporting. Full eligibility and recruitment criteria are provided within the Online Supplement. A flow diagram of infant recruitment is provided in Fig. 1. Sequence development and analysis were not determined by stream of recruitment.

### MR systems

Infants first underwent scanning on an Achieva 3T MR scanner (Philips, Best, the Netherlands) with a dedicated 32 channel receive neonatal head coil and integrated cradle system.^28^ Paired ULF scanning was then performed on a 0.064 T (64mT) Swoop® (Hyperfine, Inc., Guilford, USA–hardware version: 1.7, software versions 8.2.0 & 8.5 beta) utilising the built-in radiofrequency interference rejection method,^29^ single channel transmit/eight channel receive head coil, and infant cradle (“Baby Nest”)–see Online Supplement Figure S1. Dedicated 64mT acquisition sequences for neonatal brain imaging were optimised iteratively. FDA approved 64mT sequences, designed for adult use and provided by the manufacturer, and the optimised neonatal 64mT sequences were compared with 3T reference scans. Adverse events and difficulties in the use of the ULF system were recorded.

### Infant safety and handling

Infants referred for diagnostic scans were eligible for chloral hydrate (2,2,2-Trichloroethane-1,1-diol) sedation (50 mg/kg per orem), while imaging undertaken purely for research took place in natural sleep. All medical support, such as mechanical ventilation, intravenous infusions and/or thermoregulation were continued throughout 3T and 64mT scanning, alongside continuous monitoring of oxygen saturation, temperature, and heart rate (Phillips InVivo, Best, the Netherlands). A neonatologist supervised the clinical care of all infants during scanning sessions.

For both 3T and 64mT studies infants were swaddled using a vacuum-evacuated bag containing polystyrene beads and placed in neonatal imaging cradles designed to position the infant’s head at the magnet isocentre. Hearing protection (Dental Putty; COLTENE/Whaledent AG, Altstätten, Switzerland) and lateral immobilisation (Neonatal KC Head positioning System, Pearltec, Zurich, Switzerland) were applied prior to scanning. MR precautions were maintained for both 3T and 64mT systems, including safety screening of all staff and participants entering the MR environment, and all equipment meeting necessary MR safe or MR conditional specifications.^28^ Peak noise level inside the ULF Swoop® system is approximately 85 decibels.^30^

### MR acquisitions

#### 3T reference scans

Images were acquired using state-of-the-art protocols optimised for the Developing Human Connectome Project (dHCP),^28^ including: T1w MPRAGE with parameters TR/TE = 11/4.6 ms, TI = 713 ms, flip angle = 9°, acquired voxel size = 0.8 × 0.8 × 0.8 mm, number of slices = 135, FOV = 145 × 145 × 108 mm, SENSE factor = 1.2, bandwidth = 133 Hz/pixel, turbo factor = 121—sequence duration 4 min 35 s; and T2w imaging in the axial and sagittal planes with parameters TR/TE = 12,000/156 ms, acquired voxel size = 0.8 × 0.8 × 1.6 mm, slice gap = −0.8 mm, TSE factor = 12, bandwidth = 233 Hz/pixel, turbo factor = 12, number of slices = 125 (Axial) & 145 (Sagittal), profile order = asymmetric, sense factor = 2.1—sequence duration 3 min 12 s for each plane. Total time spent within the 3T MR scanner would be typically 45–90 min as MR protocols may include additional sequences separate from this portable imaging study (e.g., diffusion MRI, or resting state functional MRI).

#### Optimisation of 64mT MR sequences

The portable ULF system is equipped with FDA approved (all patient ages–US FDA K201722) MR sequences optimised for adult brain imaging. To optimise the system for neonatal use we developed bespoke neonatal 3D Fast Spin Echo T1 weighted (T1w) and T2 weighted (T2w) sequences. Optimisation focused on achieving isotropic resolution, enhancing tissue contrast, and maintaining signal to noise ratio (SNR). Optimisation continued iteratively leading to refinement of parameters in a progression of sequence “generations”. Images reported as optimised within this paper represent generations 5–7 of T2w sequence development, and generations 2, 3 or multi-inversion generation 4 of T1w sequence development. Individual scanning sessions may have been devoted to either T1w or T2w development, i.e., not all scans consisted of both T1w and T2w 64mT imaging. Details of the full optimisation process and technical development, including sequence durations, are provided within the Online Supplement.

#### Post-acquisition processing

For advanced 64mT sequence generations, where adequate tissue contrast had been demonstrated, we performed averaging of repeat acquisitions to enhance the SNR. To account for inter-acquisition motion, rigid body realignment was performed with sinc interpolation followed by signal averaging using FSL FLIRT and fslmaths (FMRIB, Oxford, UK).^31^^,^^32^ Masks were drawn to exclude the face and neck/chest from the alignment calculation because infant motion can generate non-rigid extra-cranial movement—see Online Supplement Figure S5. Where aligned averaging was used, the number of contributing acquisitions is listed within all figures in this paper; the total scan time of a sequence is a direct multiple of the number of acquisitions (see Online Supplement Figure S7). Final generation single acquisition duration for T2w imaging is 4 min 38 s, and for T1w imaging and myelin specific T1w imaging–6 min 55 s each.

### Radiologic assessment

All 3T images were reviewed by a specialist neonatal neuroradiologist with access to full clinical information on each participant.

Imaging from 64mT sequences was directly compared with 3T imaging without blinding in order to 1) provide rapid visual feedback on how the next sequence generation should be improved and thus plan parameter modifications using all the expertise within our group and 2) iteratively teach and inform our group how normal structures and abnormalities may appear at 64mT given nascent exposure to neonatal imaging from this novel technology.

Specifically, 64mT images were assessed for: brain tissue contrast (discernible contrast between white matter, cortical grey matter, deep grey matter, and Cerebrospinal Fluid (CSF)); brain structures (discernible gyri, corpus callosum, choroid plexi, pituitary, optic nerves, inner ear structures/course of the vestibulocochlear nerve); signal in the posterior limb of the internal capsule (PLIC) (presence or absence of T1 hyperintensity or T2 hypointensity compared with surrounding deep grey matter); and the presence of cerebral abnormalities. Examples were selected for use within this paper’s figures to demonstrate the performance of the optimised ULF system across this range of findings, alongside examples of commonly encountered ULF MR artefacts.

### Statistical analysis

Intermodal and interrater statistical analysis have not been undertaken due to the unblinded development of our sequences. Contingency table statistical analysis has not been undertaken due to the non-random nature of our convenience sampling and small number of discordant pairs.

### Role of the funding source

The funders of the study had no role in study design, data collection, data analysis, data interpretation, or writing of the report. PC, FP, TA, & ADE have access to and verified the full dataset. ADE had final responsibility for decision to publish.

## Results

### Participants

102 paired 64mT and 3T scans in 87 infants (1.17 paired scans per infant), 54 male, 33 female, were acquired either on the same day (82 scans) or within 1–31 days (median 4.5) of the 3T scan (20 scans). Median gestational age at birth was 38^+2^ weeks (range 25^+3^–42^+1^). Median postmenstrual age at scan 39^+6^ weeks (range 31^+3^–53^+4^) and median weight at scan was 3.16 kg (range 1.33–6.06)—both including follow-up scans. Two 64mT scans were abandoned early in sequence acquisition due to the infants waking, giving a 98% first time success rate; both abandoned scans were successful on second attempt on the same day. Median total acquisition time for 64mT imaging was 31 min (Interquartile range: 28–32).

67 MR examinations were carried out because of risk or suspicion of cerebral abnormality, and 35 scans were performed in infants without suspected neurological abnormality. Infants had a range of intensive care requirements and these were maintained in all cases on both MR systems: 7 were undergoing mechanical ventilation; 11 low-flow nasal oxygen supplementation; 1 continuous positive airway pressure; 8 were receiving intravenous prostaglandins to maintain patency of the ductus arteriosus due to congenital heart lesions; 20 were nursed on a temperature-controlled mattress; 2 infants had indwelling arterial cannulae, and 2 infants were receiving inotropic therapy. Co-morbidities included prematurity, pre-operative and post-operative congenital cardiac lesions, hypoxic-ischaemic encephalopathy (HIE), and developmental abnormalities.

No adverse events were recorded during any of the scanning sessions. Participant characteristics at 64mT scanning are provided in Table 1 and Online Supplement Table S1.

**Table 1:** Table of participant characteristics.

Participant characteristic	First 64mT Scan *n* = 87
Gestation at birth [Weeks^+days^]	38^+2^ (33^+3^–39^+1^)
Weight at birth [kg]	2.92 (2.03–3.45)
Age at scan [Days]	9 (4–36)
PMA at scan [Weeks^+days^]	39^+6^ (37^+4^–40^+6^)
Weight at scan [kg]	3.03 (2.45–3.45)
**Sex**	
Female (%)	33 (38)
Male (%)	54 (62)
**Medical support during scan**	
Mechanical ventilation (%)	7 (8)
CPAP (%)	1 (1)
LFNC (%)	9 (10)
Prostaglandin infusion (%)	8 (9)
Temperature controlled mattress (%)	19 (22)
Inotropic therapy (%)	2 (2)
Arterial line (%)	2 (2)
Given chloral hydrate sedation^a^ for 3T MR scan (%)	29 (33)
**3T scan indication**	
Clinical	59 (68)
Infants without suspected neurological abnormality	28 (32)
**Co-morbidity**	
Born preterm (%)	30 (34)
Congenital cardiac lesions (%)	23 (26)
Encephalopathy, abnormal movements/seizures or suspected HIE (%)	17 (20)
Suspected developmental brain abnormalities (%)	10 (11)
Suspected metabolic disorder (%)	2 (2)
Post-meningitis (%)	1 (1)
Post-ECMO (%)	1 (1)
**Focus of sequence development**	
Solely T1 weighted sequence development (%)	44 (51)
Solely T2 weighted sequence development (%)	9 (10)
T1 & T2 weighted sequence development (%)	34 (39)

Continuous data are shown as: Median (Interquartile range).

PMA: post-menstrual age; CPAP: continuous positive airway pressure; LFNC: low flow nasal cannulae (Oxygen); HIE: hypoxic ischaemic encephalopathy; ECMO: extra-corporeal membrane oxygenation.

a No infants without suspected neurological abnormality received chloral hydrate in this cohort.

### ULF portable MR imaging

**Fig. 2:**
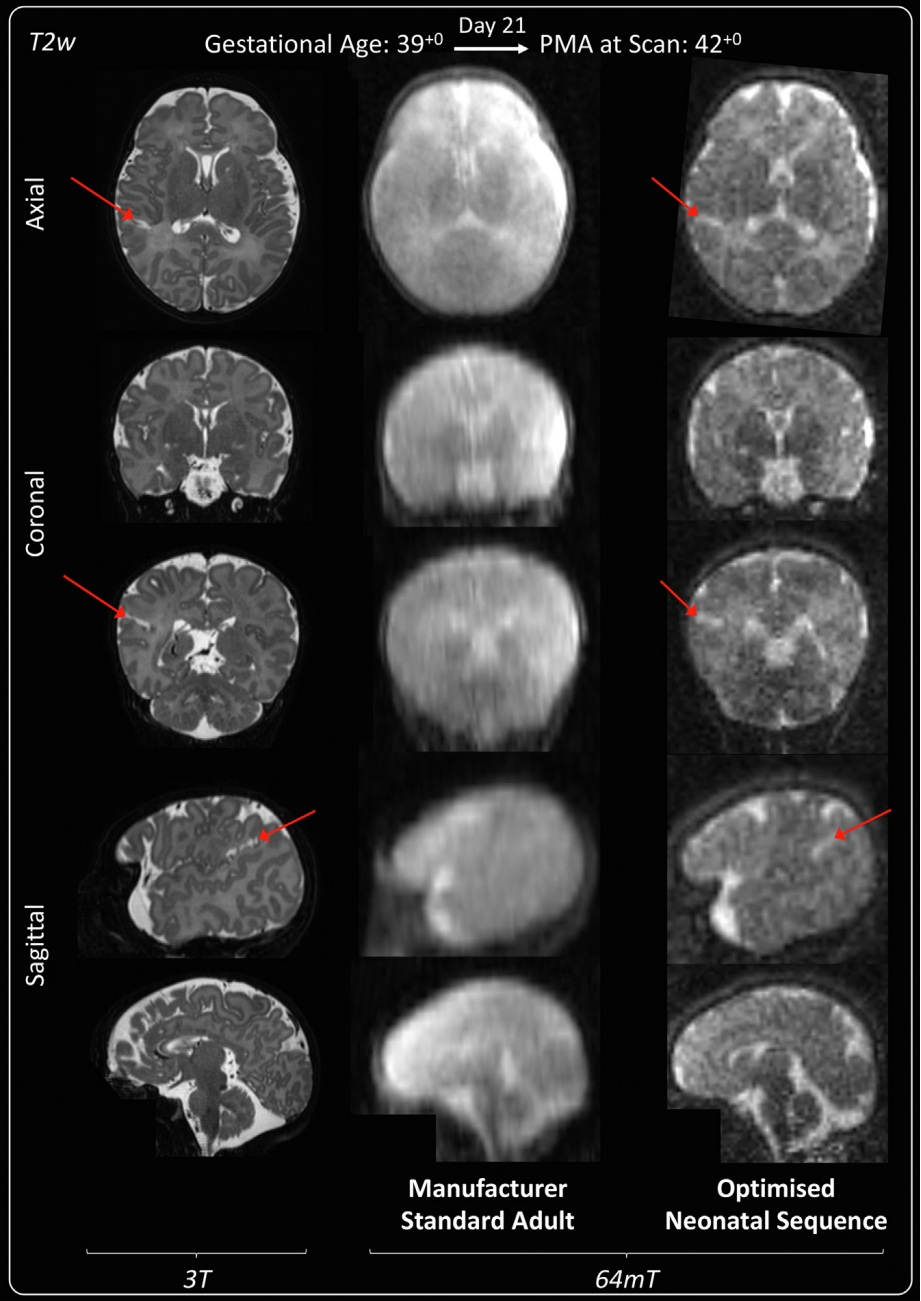
**T2 weighted manufacturer standard adult 64mT sequence compared with reference 3T imaging and dedicated 64mT neonatal sequence.** Term infant with small right-sided focal cortical infarction in the superior parietal lobe (red arrows). 3T MR scan was performed on day 15 and 64mT scan on day 21. 64mT adult sequence shows poor tissue contrast, the cortical infarct is not identifiable and there is asymmetric signal inhomogeneity (generalised left sided high T2). Subtle abnormal cortical signal is present on the optimised 64mT neonatal sequence in the location of the infarct. Deep grey matter on axial plane and cerebellum on coronal plane remain unclear and PLIC not identified due to persistent noise and narrow contrast. PMA: post-menstrual age; PLIC: posterior limb of the internal capsule.

Manufacturer standard adult T1w inversion recovery imaging at 64mT was unable to differentiate neonatal cortex, white matter, deep grey matter, or ventricles in all 4 infants for which it was tested, and thus provided insufficient clinical information for the developing neonatal brain; example imaging is shown in Online Supplement Figure S3. Additionally, the longer T1 of unmyelinated white matter causes this tissue to still be negative at the inversion time used for adult imaging, and there are many signal cancellation artefacts where the signal sign changes. Manufacturer standard adult T2w imaging at 64mT (Fig. 2) also poorly differentiated neonatal cortex and white matter in all 3 infants for which it was tested. Bespoke neonatal 64mT imaging and high field 3T images are also shown in Fig. 2 for reference.

#### Optimised 64mT images

As shown in Table 2, our multi-acquisition signal averaged T1w (3× acquisitions) and T2w (≥5 acquisitions) neonatal optimised sequences successfully produced imaging with sufficient contrast to differentiate the ventricular CSF, white matter, and grey matter in 7/7 infants for T2w imaging, and 10/11 infants for T1w imaging. Key macro-structures were identifiable–such as the optic nerves (T2w: 7/7 infants, T1w: 10/11 infants), choroid plexi (T2w: 7/7 infants, T1w: 8/11 infants) and corpus callosum (T2w: 7/7 infants, T1w: 6/11 infants).

**Table 2:** 64mT visual tissue/structural performance of our latest multi-acquisition T2w (Minimum of five 1.75 mm isotropic acquisitions) and multi-TI T1w imaging strategy (3 × 2 mm isotropic acquisitions of TI 400, 500 & 600 ms).

	T2w neonatal optimised sequences (Generation 7 with minimum 5 acquisitions for signal averaging) n = 7 infants (%)	T1w neonatal optimised multi-TI imaging strategy n = 11 infants (%)
Structures visualised		
Optic nerves	7/7 (100)	10/11 (91)
Inner ear structures	3/7^a^ (42)	0/11 (0)
Vestibulocochlear nerve	1/7^a^ (14)	0/11 (0)
Pituitary	5/7 (71)	6/11 (55)
Choroid plexi	7/7 (100)	8/11 (73)
Ventricles	7/7 (100)	11/11 (100)
Corpus callosum	7/7 (100)	6/11 (55)
Tissues visualised		
PLIC myelin signal	4/7 (57)^b^	10/11 (91)
Cortex	7/7 (100)	10/11 (91)
Deep grey matter	7/7 (100)	10/11 (91)
White matter	7/7 (100)	10/11 (91)
Gyri	7/7 (100)	4/11 (36)

Percentage measure of number of infants for which tissue/structure is observable and distinguishable from adjacent tissue types.

PLIC: posterior limb of the internal capsule; TI: inversion time.

a Cause of failure was obfuscation of the vestibulocochlear nerve and inner ear structures by zipper artefact in 4 infants.

b 3 out of 4 signal congruent sequences were congruent in infants without myelin signal (i.e., normal preterm or abnormal term).

The pituitary gland (T2w: 5/7 infants, T1w: 6/11 infants), inner ear structures (T2w: 3/7 infants, T1w: 0/11 infants) and vestibulocochlear nerves (T2w: 1/7 infants, T1w: 0/11 infants) were less consistently identified in these sequences, with obfuscation of the vestibulocochlear nerve and inner ear by MR zipper artefact occurring on T2w imaging in 4/7 infants.

Enhanced interference removal is now available from the manufacturer which ameliorates zipper artefacts resulting from external environment radio-frequency noise; in a sub-cohort of infants with optimised 64mT T2w scans using this new software (all limited to only 1 or 2 acquisitions for averaging) the vestibulocochlear nerve was visualised in 5/8 infants and the inner structures visualised in 7/8 infants—see also Artefact *section*.

**Fig. 3:**
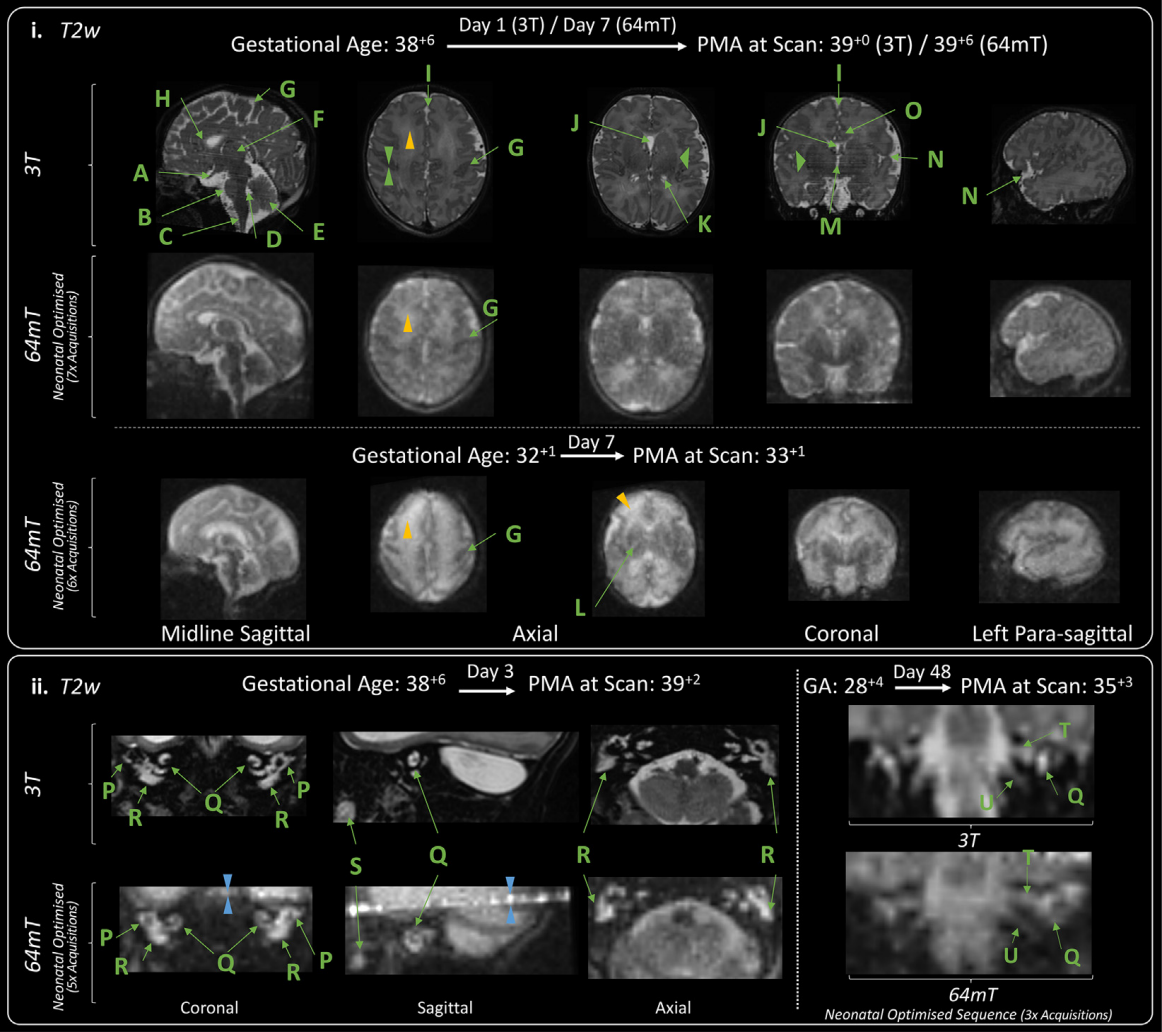
**Neuroanatomy and structural imaging.** (i) Example T2w imaging of the normally developed term and preterm brain. Green Double Arrowheads—Cortex. Green Single Wide Arrowheads—Deep Grey Matter. Amber Single Narrow Arrowhead—white matter (higher T2 signal is apparent in the preterm infant). Deep Grey Matter and medial temporal lobes appear as lower T2 intensity in comparison with 3T imaging. Labelled structures are visible on both 3T and 64mT imaging: A) Optic Nerve, B) Pons, C) Medulla, D) Fourth Ventricle, E) Cerebellar Vermis, F) Thalamus, G) Central Sulcus, H) Anterior Corpus Callosum, I) Interhemispheric Fissure, J) Cavum Septum Pellucidum, K) Lateral Ventricle, L) Internal Capsule (Only visible in the preterm infant imaging; as high T2 streak), M) Third Ventricle, N) Sylvian Fissure, O) Cingulate Gyrus. (ii) Inner Ear Structures. Zipper artefact (Blue double arrowheads) transects the inner ear structures in some infants. As shown, inner ear structures and cranial nerves can be difficult to delineate on standard high field (3T) acquisitions, without dedicated views. P) Semi-Circular Canal, Q) Cochlear, R) Vestibule, S) Tooth; and courses of the T) Vestibulocochlear Nerve, and U) Glossopharyngeal Nerve. GA: gestational age; PMA: post-menstrual age.

Example neonatal optimised T2w 64mT imaging is provided in Fig. 3. Comparable neuroanatomical labelling is shown between optimised 64mT sequences and state-of-the-art 3T reference imaging (Fig. 3.i). Additionally, this figure shows that optimised 64mT sequences can achieve clear maturational differentiation between the term and preterm brain: characteristically, the preterm infant has high signal within the white matter and less developed gyral folding.

Exemplar inner ear imaging is shown in Fig. 3.ii—the zipper artefact in this scan missing and sitting above the well delineated semi-circular canals, cochlear, and vestibule. Further imaging is also shown delineating the course of the vestibulocochlear nerve prior to its bifurcation.

**Fig. 4:**
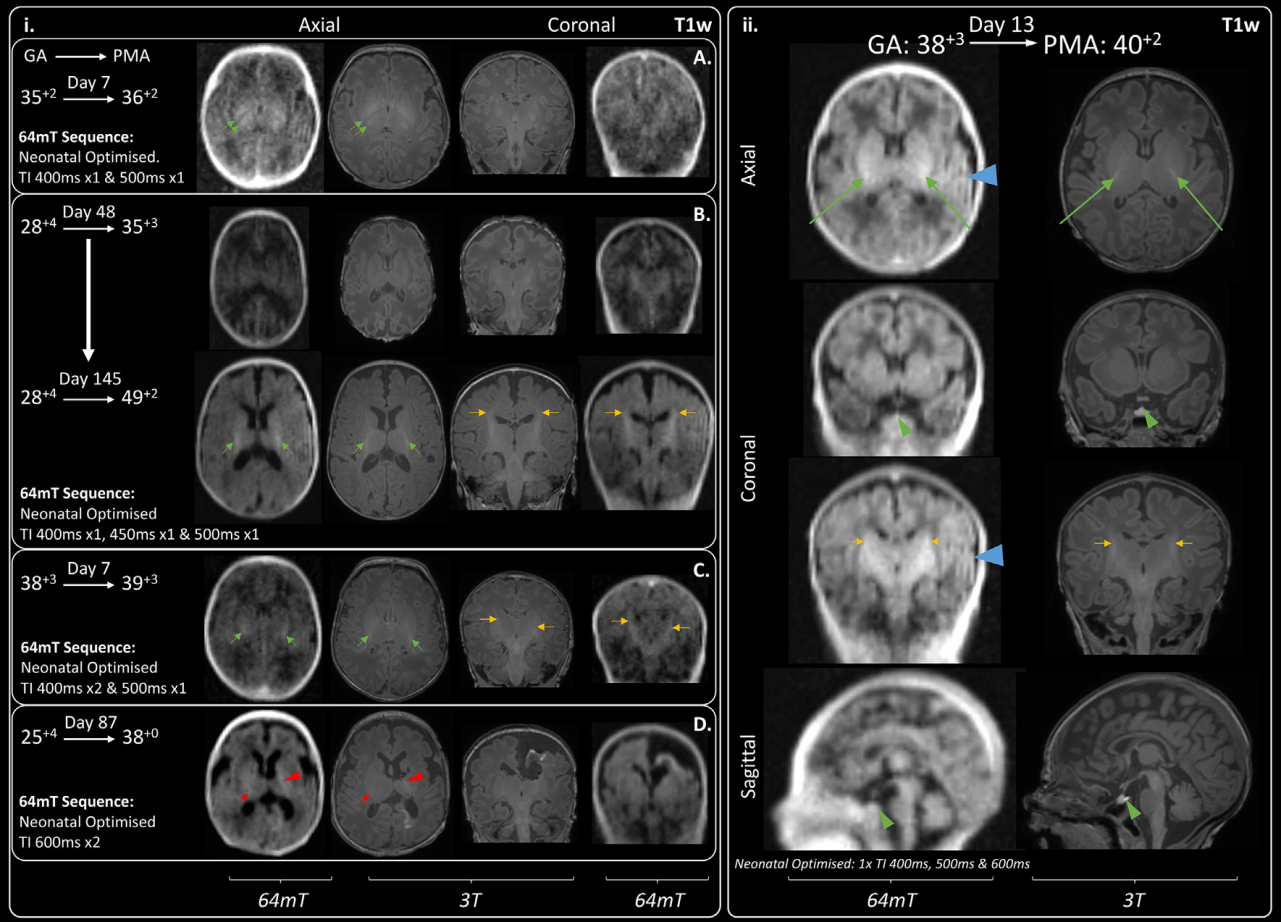
**T1 signal within the Posterior Limb of the Internal Capsule (PLIC).** Single green arrows indicate normal T1 hyperintensity within the PLIC and single amber arrows indicate normal T1 hyperintensity within the corticospinal tracts. (i) Case series demonstrating progression of T1 signal generated by myelination across range of gestations: A) Preterm infant with absence of myelin signal within the PLIC which appears hypointense due to normal for age high T1 signal within the ventrolateral nucleus posteriorly and globus pallidus anteriorly (double green arrows). B) Very preterm infant imaged during prematurity and again at post-term -demonstrating evolution of myelination of the full length of the PLIC by 49^+2^ weeks gestation. 64mT scan resolution may preclude visualisation of thin strip of T1 hyperintensity seen on 3T images within the anterior limb of the internal capsule. C) Small amount of T1 myelin signal seen within PLIC bilaterally, infant imaged just prior to their expected delivery date. D) Asymmetry in PLIC signal: small amount of T1 myelin signal on right-side (single red arrow), with abnormal high T1 signal for gestation along the left PLIC (red arrowhead) secondary to extensive left-sided haemorrhagic infarction. (ii) Exemplar 64mT T1w neonatal imaging—multi-inversion time sequence combination facilitates superimposition of PLIC signal alongside white matter, grey matter, and CSF contrast. Pituitary tissue is labelled with a green arrowhead. Ghosting artefact is evident on left-side (blue arrowhead), this requires further investigation. GA: gestational age; PMA: post-menstrual age.

Examples of 64mT imaging of the pituitary are included in Fig. 4.ii and Online Supplement Figure S16. Similar to 3T, pituitary tissue, including the infundibulum, appears T2 hypointense (dark) against T2 hyperintense (bright) CSF on T2w sequences. On T1w images, the posterior pituitary tissue is hyperintense, however, this is indistinctly seen at the limit of our current T1w sequence resolution.

Imaging of the PLIC, and myelin-like signal within the PLIC, required dedicated sequence development. In infants undergoing manufacturer standard or our first generation neonatal T1w sequences PLIC signal congruence was achieved in 1/7 infants (14%). Infants who underwent our most advanced 64mT T1w sequences demonstrated 64mT-3T PLIC signal congruence in 10/11 cases (91%); shown in Table 2 and Fig. 4.ii. Examples of characteristic visual T1 signal evolution within the anatomical location of the PLIC, as post-menstrual maturity increases, are provided in Fig. 4.i. Progression from an iso- or hypo-intense PLIC in prematurity, to a defined T1 hyperintensity consistent with emerging myelination in term and post-term maturity is shown. Furthermore, asymmetrical appearances can be seen in response to lateralised pathology in an infant with unilateral haemorrhagic infarction leading to abnormal excessive high-signal mimicking myelin and not confined to the PLIC (Fig. 4—case D). PLIC T1 signal may be indistinct (Fig. 4—case C) or even appear as exaggerated against 3T imaging (Fig. 4—case B at post-term).

The visual structural performances of our earlier generation neonatal T1w and T2w 64mT sequences are summarised in Online Supplement Tables S5 and S9; and further breakdown of 64mT T1w PLIC signal congruence is provided in Online Supplement Tables S6–S9, and in Online Supplement Figures S10 and S11.

### Unblinded comparison of ULF MR with 3T: clinical performance

Most reference 3T MR brain scans within this study identified at least one abnormality and were thus classified as ‘abnormal’; this ranged from clinically insignificant to diagnosis and treatment defining pathology.

In the 34 infants who underwent paired optimised T2w 64mT and 3T reference scanning, 29 3T reference scans were identified as abnormal, compared with 26 of the 64mT T2w scans, with no false positive 64mT images. Similarly, in the 30 infants who underwent paired optimised T1w 64mT and 3T reference scanning, 23 3T T1w reference scans were identified as abnormal, compared with 16 64mT T1w scans, with no false positive 64mT images. ULF MRI thus provided an abnormal result in 90% of 64mT T2w and 70% of T1w brain imaging where at least one known abnormality was present. In the three ‘false-normal’ 64mT T2w scans, missed pathology consisted of small white matter punctate lesions and small periventricular cystic lesions. Of the seven ‘false-normal’ 64mT T1w scans, missed pathology consisted of the same periventricular cysts, small subependymal cysts, small high T1 linear abnormalities within the thalamus, products of an old germinal matrix haemorrhage and white matter punctate lesions.

**Fig. 5:**
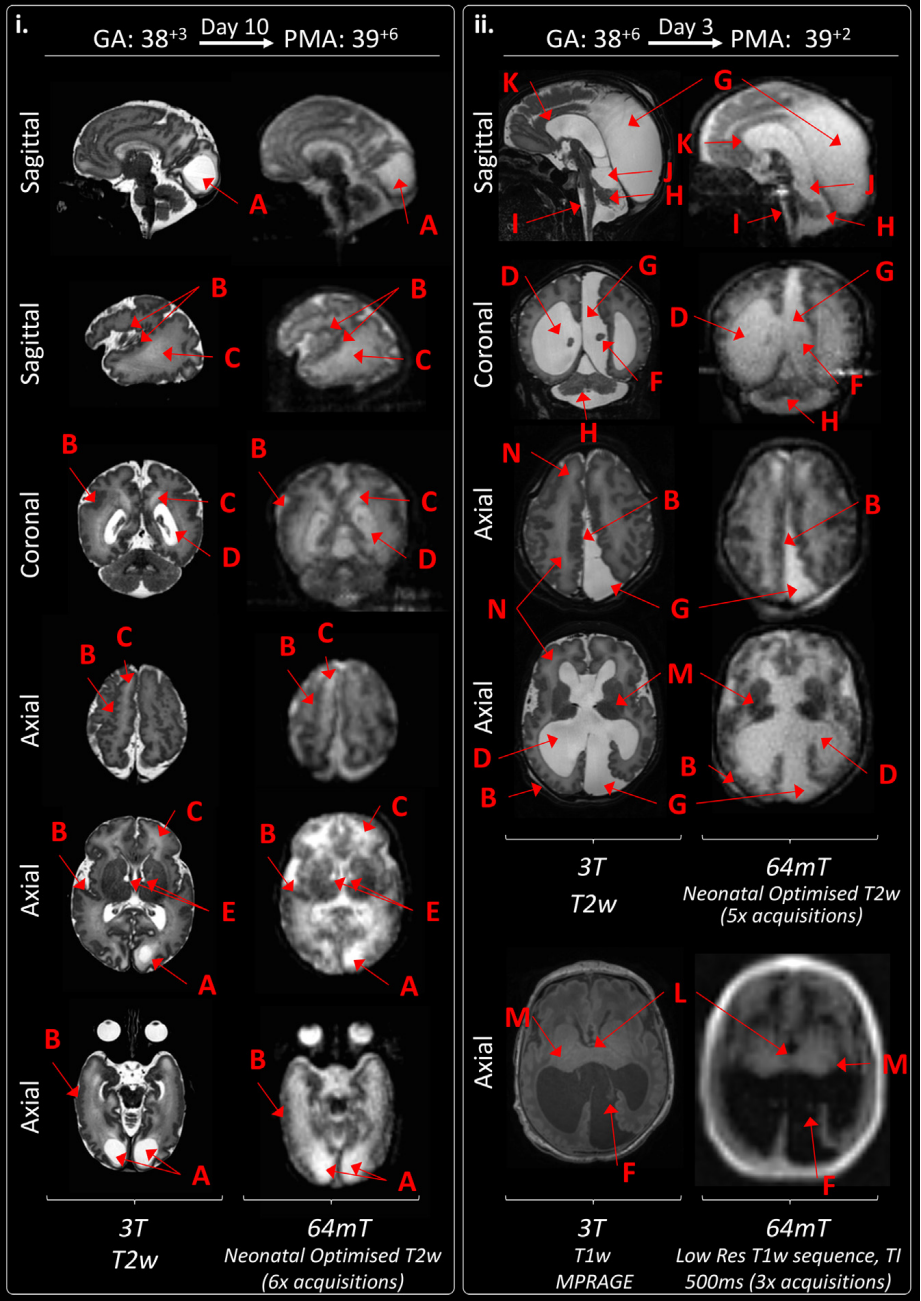
**Congenital structural anomalies and disrupted development.** (i) In utero CMV infection leading large bilateral sub-occipital cysts (A), extensive polymicrogyria (some examples are given—B—depicted on 64mT as thickened cortex with shallow sulci), patchy WM T2 hyperintensities (C), ventriculomegaly (D), and bilateral subependymal cysts (E). (ii) Cerebellopontine Hypoplasia with severe ventriculomegaly (D) and malpositioned choroid plexi (F), leading to enlargement of a midline crossing inter-hemispheric cyst (G). The left cerebral hemisphere is smaller than the right, there is extensive polymicrogyria (examples given—B), hypoplastic cerebellar hemispheres and vermis (H), hypoplastic pons and brainstem (I), dilated quadrigeminal cistern (J), thin stretched corpus callosum (K), small deep grey matter structures with wide thalamic adhesion (L) and absence of myelin which is abnormal for this infant’s gestational maturity (M). White matter striations are seen on 3T T2w imaging (N)—some correlative findings are present on 64mT imaging, but these are ill-defined, presenting mainly as generalised high T2 white matter at ULF. Choroid plexi are markedly less distinct, but remain identifiable, on 64mT imaging. GA: gestational age; PMA: post-menstrual age; Low Res: low resolution.

The majority of abnormal scans identified multiple pathologies, again non-identified pathologies on neonatal optimised 64mT sequences tended to be small or subtle lesions, particularly within the white matter such as small punctate lesions, striations, and microcysts (Table 3), which may be of uncertain prognostic importance particularly in the context of other major brain pathology (see examples in Fig. 5.ii, and Online Supplement Figures S12–S14). Pathologies not identified on one tissue contrast may still be seen on other contrasts, for example, polymicrogyria was not seen on T1w sequences but was identifiable on T2w sequences.

**Table 3:** Clinical performance of 64mT T2w and T1w imaging in unblinded comparison with abnormalities identified on 3T reference imaging.

Pathology identified	T2w paired imaging Infants n = 34			T1w paired imaging Infants n = 30		
	64mT	3T	64mT/3T congruence (%)	64mT	3T	64mT/3T congruence (%)
**Acquired abnormalities (Including acute and/or chronic injuries)**						
Cortical infarction	6	6	100	4	4	100
White matter infarction	6	6	100	4	4	100
Intraventricular haemorrhage	9	10	90	5	6	83
White matter cysts	3	5	60	0	1	0
BGT cysts	0	1	0	0	1	0
Generalised high T2 signal in white matter (Very/Extremely preterm infant)	2	2	100	NA		
Generalised high T2 signal in white matter (Preterm infant at TEA)	2	2	100	NA		
Abnormal low T2/high T1 signal in BGT (Consistent with clinical context of HIE)	2	2	100	1	1	100
Abnormal high T2/Low T1 signal in white matter (Consistent with clinical context of HIE)	2	2	100	1	1	100
Cortical T1 highlighting	NA			1	1	100
Increase T1 signal in dentate nucleus	NA			0	1	0
Extra-cerebral haemorrhage	1	1	100	2	2	100
Subdural haemorrhage	4	7	57	8	9	89
Punctate white matter lesions	0	4	0	0	9	0
White matter haemorrhagic foci	1	2	50	0	0	–
Cerebellar microhaemorrhages	0	4	0	0	3	0
Patchy high T2/low T1 white matter	2	2	100	1	1	100
Sub-ependymal cysts	3	3	100	0	1	0
Linear T1 abnormalities in thalamus (Possibly relating to a past vasculitic process)	NA			0	1	0
Ventricular dilatation, prominence or asymmetry (Could be acquired or developmental)	12	12	100	7	8	88
**Abnormalities in development or disrupted fetal development**						
Hypoplastic cerebellar vermis	3	3	100	2	2	100
Hypoplastic cerebellar hemisphere	1	1	100	0	0	-
Dysplastic cerebellar foliation	0	1	0	0	1	0
Hypoplasia of brainstem structures	3	3	100	2	2	100
Increased extra-axial space/Widened interhemispheric fissure	7	7	100	6	6	100
Colpocephaly	2	2	100	2	2	100
Enlarged cisterna magna	2	2	100	2	2	100
Polymicrogyria	3	3	100	0	1	0
Agenesis of corpus callosum	2	2	100	2	2	100
Abnormal hippocampal formations	1	1	100	0	1	0
Abnormal white matter striations	0	1	0	0	0	–

Optimised T2 weighted imaging (generation 5–7) and optimised T1 weighted imaging (Generations 2, 3 and multi-inversion generation 4) only.

BGT: basal ganglia & thalamus; TEA: term equivalent age; HIE: hypoxic ischaemic encephalopathy; NA: not applicable.

The performances of optimised T2w and T1w 64mT sequences in corroborating pathologies identified at 3T are listed in Table 3.

Unoptimised 64mT sequences have not been extensively tested against major pathologies in this series, as, once created, optimised sequences were used in preference. However, a full list of 3T congruous and non-identified abnormalities on unoptimised and optimised 64mT sequences, for both T1w and T2w imaging, is provided in Online Supplement Tables S10 and S11.

#### Congenital brain dysgenesis

As shown in Table 3, neonatal optimised ULF structural definition was sufficient to detect major developmental abnormalities such as hind-brain hypoplasia (Hypoplasia of: vermis 3/3 infants on T2w & 2/2 infants on T1w, cerebellar hemisphere 1/1 infant on T2w, and brainstem structures 3/3 infants on T2w & 2/2 infants on T1w), agenesis of the corpus callosum (2/2 infants on T1w & T2w) and, on T2w imaging, polymicrogyria (3/3 infants on T2w). Examples of such congenital brain dysgenesis are shown in Fig. 5. Key clinical information, which may suggest aetiology and prognosis, such as large cystic change, polymicrogyria, ventricular dilatation, and pattern of gyral folding are identifiable in these image-sets. Further examples of developmental abnormalities are provided in Online Supplement Figures S17–S18.

#### Perinatally-acquired brain injury

**Fig. 6:**
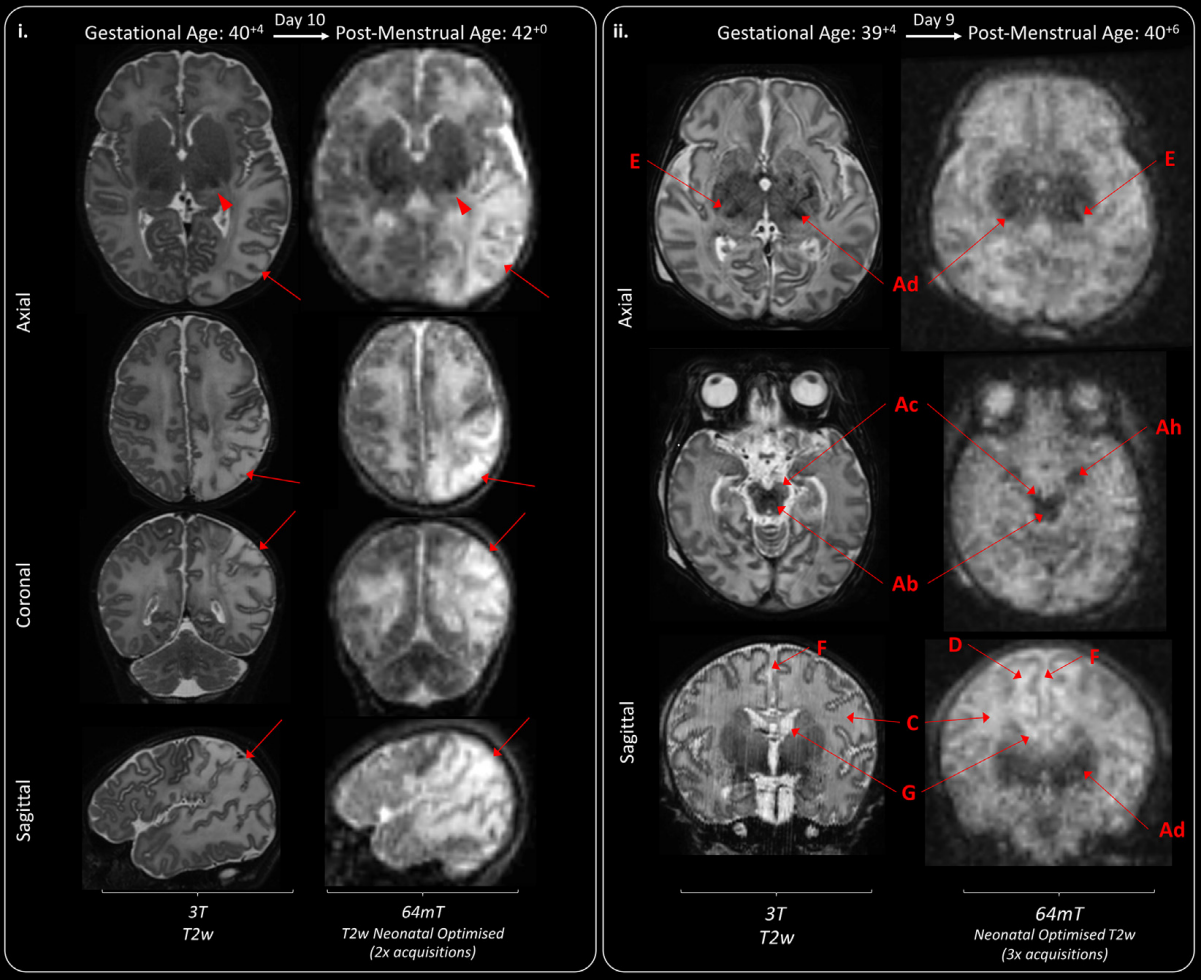
**Acquired injury in the normally formed brain.** (i) Diffuse left sided white matter and cortical parieto-occipital infarction (red arrows). High T2 signal is also seen within the left posterior thalamus, suggestive of secondary network injury (red arrowhead). A video of this study is available as an Online Supplement. (ii) Bilateral basal ganglia, thalamic, cortico-spinal tract, and mesencephalon injury secondary to perinatal hypoxia-ischaemia. Hypoxic-ischaemic injury revealed as abnormal low T2 within the deep grey matter (Ad), cerebral peduncles (Ac), hippocampi (Ah) and brainstem (Ab). There is diffuse abnormal high T2 signal in the white matter (C), with areas of sub-cortical white matter highlighting on 64mT T2w images (D). The Posterior Limb of the Internal Capsule is abnormally T2 hyperintense -abnormal for gestational maturity (E). Additional findings include widened interhemispheric fissure (F), and bilateral sub-ependymal cysts (G).

MR signal abnormalities consistent with major acquired tissue pathology such as cortico-white matter infarction (6/6 infants on T2w & 4/4 infants on T1w) and hypoxic-ischaemic injury of the cortex (1/1 infant on T1w), white matter (2/2 infants on T2w & 1/1 infant on T1w) and deep grey matter (2/2 infants on T2w and 1/1 infant on T1w) were detectable on optimised 64mT imaging (Table 3). As shown in the examples in Fig. 6, patterns of abnormal high and low MR signal intensity, denoting areas of injury, are similar to the characteristic appearances expected in high field MR imaging. Acute infarction presenting as high T2 and low T1, acute haemorrhage as high T1 and low T2, and acute ischaemic injury to the deep grey matter structures as low T2 and high T1. The examples in Fig. 6 also demonstrate that the signal contrast of areas of injury identified on 64mT imaging may be more extreme than seen on the corroborating 3T imaging. T2w imaging of the neonatal stroke is also available to view as the Video Online Supplement.

#### Artefacts

To some degree, ‘zipper’ artefacts due to unresolved external radio-frequency noise were seen in most images with varying degrees of severity. This zipper partially obscured anatomy–areas at the periphery of field of view were most affected, such as inner ear structures, vestibulocochlear nerve, and pituitary. Examples are annotated in Fig. 3.ii, and Online Supplement Figures S3 and S12. Zipper artefact has improved with enhanced interference removal through manufacturer software advances. Significant zipper was present in 44/46 of the 64mT T2w sequences, and all 64mT T1w sequences, performed prior to this software update. Following the update, the zipper was absent in 2/8, and only subtly present in 6/8 of the T2w sequences and absent in all 36 T1w sequences.

Left-sided field inhomogeneity, with artefactual asymmetrical high T2 signal appearing on the participant left, impacted early generation T2w sequences (all 4 iterations of the manufacturer standard adult 64mT sequences, all 3 iterations of the manufacturer non-standard neonatal 64mT sequence and 1 third generation neonatal sequence), resolving in prominence with more optimal tissue contrast in all later generation neonatal sequences. An example of artefactual asymmetrical high T2w signal may be seen in the manufacturer standard adult 64mT sequence shown in Fig. 2. When present, motion artefact led to non-differential blurring of all tissues—examples are shown in the Online Supplement Figures S10 and S15. This may be subtle and could lead to unrecognised masking of pathology, as well as loss of definition of slight tissues and anatomical features which are also susceptible to noise.

Low SNR imaging can also lead to confusing hyperintensities in all contrasts and, in particular, visual loss of SNR occurs towards the opening of the head coil and may lead to loss of imaging quality of the posterior fossa (e.g., absence of cerebellar detail on coronal slice in Fig. 2) or of finer brain structures such as the pituitary, optic nerves, vestibulocochlear nerves and cortex.

Additionally, the medial temporal lobes/hippocampi demonstrate relatively low T2 or high T1 signal on 64mT imaging in the absence of pathology on corresponding 3T imaging. This may be reflective of partial voluming in an area of tight cortical folding, though is also seen within the central deep grey matter structures and could be misconstrued as pathology to reporters unaware of these appearances (notable examples are seen on coronal slices in Figs. 2 and 3.i).

## Discussion

MR Brain Imaging has many important clinical and research applications in infants. However, MR imaging is complex, expensive, and often not available outside advanced medical facilities. As a result, access to MRI is 140-fold less in low-income countries (0.188 MRI units per million population) compared with high income countries (26.529 MRI units per million population), and there remain countries for which there is no access to MRI at all.^33^ Worldwide, the majority of infants at risk of brain pathology thus do not have access to optimum clinical neuroimaging. Even within high-resource settings access is limited as radiology departments typically face high demand relative to availability. Additionally, the need to transport patients to imaging facilities may preclude MR scanning for the sickest and most vulnerable infant, in whom neuroimaging may be most valuable to guide management. The ability to distinguish underlying pathology is critical in the diagnosis of infants presenting with neurological abnormality; as clinical examination alone may be unable to differentiate between a wide range of aetiologies, with each potentially requiring different management.

FDA approved 64mT MR sequences designed for adult use showed limited visual brain tissue contrast and thus highlight a need for optimised neonatal-specific MR sequences.^20^^,^^21^^,^^23^ Following iterative and explorative parameter optimisation, neonatal-optimised MR sequences enhance visual tissue contrast. We have shown in a number of examples that optimised 64mT sequences can identify and differentiate key brain tissues and neuroanatomical structures such as the optic nerves, inner ear, course of the vestibulocochlear nerve, pituitary tissue, anatomically identifiable gyral folds and T1 signal within the PLIC. These structures are frequently assessed when developmental or acquired brain abnormalities are suspected. We also demonstrate in unblinded analysis that ULF MR can detect important diagnostic congenital abnormalities including absent corpus callosum and polymicrogyria, as well as areas of brain injury from infarction or perinatal hypoxia-ischaemia—each have clear implications for clinical management.

ULF neonatal optimised sequences thus demonstrate potential for clinically actionable findings: many aspects of our investigation such as the appearance of the PLIC, basal ganglia and cortex are highly relevant, commonly impacted by pathology, and the precise diagnostic, aetiological, and prognostic information these provide at high-field MR imaging are already used to 1) inform compassionate decision making in infants receiving life-sustaining treatment—where that treatment may be futile, 2) guide genetic, infective or metabolic investigation–where these pathologies were not previously suspected, and 3) inform clinicians who need to both plan follow-up with limited resources as well as counsel parents as to their infant’s future.^2^^,^^9^^,^^11^

Furthermore, neurosurgical planning and monitoring could also potentially be enhanced by portable MR systems—given its ability to be taken to the cotside of critically unwell infants and the ease at which it could be used to produce serial imaging with high temporal resolution of evolving pathology. Whilst bedside neonatal Cranial USS provides excellent assessment of intra-ventricular haemorrhage, ventricular dilatation and cysts, portable MR has the potential advantage of providing whole cranium 3D acquisition, not limited by the location of the fontanelles as USS optic-windows. 3D acquisitions may thus provide additional clinically actionable information such as measured fluid volumes (not just 2D indices) and potentially identify areas of extra-cerebral intra-cranial haemorrhage which may be missed on USS, and currently require irradiating Computed Tomography (CT) brain imaging out-of-hours in many hospitals.^13^^,^^34^ The optimised system is now ready to be tested in a formal validation study against high quality Cranial USS, CT, and high-field MR for such applications.

Our 64mT sequences did not perform well at identification of small foci such as microhaemorrhages, small cysts and white matter punctate lesions. It is important clinicians are aware of potential limitations of this technology as these findings may still bear prognostic relevance and thus still necessitate the current practice of complimentary use of MRI with ultrasound; which performs well in identification of large cysts.^13^^,^^35^^,^^36^ In addition, we suspect these 3D fast spin echo sequences are highly susceptible to motion corruption with risk of masking of pathology and inconsistency in anatomical and tissue definition; which may explain variation in quality of imaging and structural delineation.

Within our series, ULF imaging is feasible and achievable with imaging times of approximately 30 min. Whilst repeated acquisition benefits SNR it has the disadvantage of increasing total scan duration. Further pulse-sequence modification (e.g., turbo factor), and development of bespoke neonatal head coils, may be able to shorten scan times further, or indeed be used to maintain current scanning times in the pursuit of even higher resolution imaging to overcome current limitations in white matter definition. Ultimately the balance between scan time and acceptable level of SNR may be determined by scan indication or study question. Of note, our current pipeline for multi-acquisition registration and signal averaging requires manual user operation and would thus be impractical in a clinical context. Automation of this process is feasible and required before dissemination for widespread use.

Whilst visualisation of structures such as the pituitary and inner ear is possible on 64mT neonatal optimised brain sequences, even at high field, dedicated high resolution inner auditory meatus sequences and small field of view, contrast enhanced, pituitary sequences may be required for full diagnostic evaluation.

There are also some limitations to this study. Given the unblinded nature of our optimisation, it is not possible to provide estimates on imaging sensitivity, specificity, and false positive/negative values. Unblinded visual comparison could have created bias to interpretation of 64mT imaging and thus positive findings that we have annotated and recorded on 64mT imaging may not have been identified without comparative 3T imaging. Similarly, bias could have prevented artefact from being falsely identified as pathology on 64mT due to the presence of reassuring ‘normal’ appearances on 3T imaging. Clinical reporting of lower resolution, higher noise and artefact impacted imaging may risk both masking or mimicking of pathology and thus requires new skill-sets and experience beyond that of interpreting high-field MR imaging.

Although we have optimised two complimentary MR structural contrasts; T1w and T2w, we did not investigate or optimise FLAIR or diffusion MR acquisitions which are also possible at 64mT. Diffusion sequences are an important part of current high-field MR protocols but require further development at ultra-low field before they are ready for neonatal testing. Additionally, modalities such as MR spectroscopy, susceptibility weighted imaging, venograms and arteriograms are currently not available at ULF and require technical development.

We used oral sedation for clinically indicated 3T imaging but never for 64mT imaging in isolation, and we always performed 64mT after 3T imaging—our study design thus risks misclassification bias, with a bias of motion corruption against 64mT imaging.

Benefits of this novel technology include the manufacture of low-cost MR scanners through both commercially available and open-source domains, creating the potential for a shift-change from high-cost low-access to high-access MR brain imaging.^14^^,^^17^^,^^37^ Serial at-the-bedside MR scanning could facilitate understanding of brain growth and development in relation to the extra-uterine environment, benefit infants undergoing intensive care who are too sick to transport to radiology departments, may aid implementation of future novel time-critical but pathology-specific neuroprotective treatments, and MR scanner access could reach areas of the globe for which there is currently no provision. Blinded validation studies are required to establish if optimised neonatal ULF imaging can meet this potential. If it can, many strategic and logistical challenges, such as the provision and training of many more clinicians enabled in neonatal ULF neuroradiological reporting will need to be met for successful implementation. Furthermore, sites undertaking portable neonatal MR imaging will need reliable and reproducible means for infant positioning–to ensure comfort and minimise motion disruption of acquisitions. This should be a due consideration prior to deployment of this portable technology. We have used vacuum-evacuated bags and inflatable ear cuffs; alternative positioning aids could include swaddling with sheets or blankets and small pillows or pads.

With the use of neonatal optimised pulse-sequences, this technology is now ready for these further studies to assess diagnostic efficacy, qualify 64mT imaging as a biomarker for neurodevelopmental outcomes and perform health-economic evaluation in the neonatal population globally, across all ethnicities. Studies are required both in low-resource settings as well as in high-resource clinics/intensive care units where other neuroimaging tools are currently available.

ULF MR systems could be transformative and provide equity in neonatal clinical care and neuroimaging research–but expert dedicated technical development, validation and implementation are mandatory.

## Contributors

PC wrote first draft of manuscript, performed the literature search, prepared manuscript figures, supervised all scans, post-processed all MR sequences, and undertook visual assessment of all 64mT imaging. FP, PC & JVH planned 64mT sequences to meet clinical and research goals. FP undertook programming of all development and optimised 64mT sequences with contributions from RT. PC, FP, DC, SW, SC, TA, MAR, JVH & ADE planned imaging goals and regularly reviewed imaging data to guide recruitment, sequence development and analysis. PC, DC, & JA recruited and consented for MR scans. PC, FP, JA, MM, TA, JVH & ADE devised operating procedure for safe use of 64mT system. FP prepared MR local rules for use of the 64mT system. AU contributed intellectually to image registration and averaging procedures. JO’M contributed intellectually to inter-acquisition registration and motion correction. MAR reviewed reference 3T imaging. TA is study PI, ADE study CI and statistician. SW led the conceptualisation and funding acquisition of the wider, Ultralow field Neuroimaging In The Young (UNITY) programme. All authors contributed intellectually to revision of drafts and approve the final submission. PC, FP, TA & ADE have accessed and verified the underlying imaging data and are data guarantors.

## Data sharing statement

Deidentified MR data is available to external investigators, on valid request, through secure electronic data-sharing platforms. All requests will require legitimate scientific purpose, and will require signed agreement to acknowledge data source, agreement not to perform secondary data sharing without prior approval and agreement not to reverse engineer data to attempt to de-anonymise participants. Data acquired through ethics approval 19/LO/1384 will not be transferred outside the European Economic Area.

## Declaration of interests

PC is supported by the Medical Research Council Centre for Neurodevelopmental Disorders [MR/N026063/1] to undertake this work and received an educational stipend from ISMRM to attend the international ISMRM conference and present this work. FP was employed by the Guy′s & St. Thomas′ NHS Foundation Trust & King’s College London as a senior MR physicist at study outset, during experimental design and initial participant recruitment, he is now a senior clinical scientist employed by Hyperfine Inc., since May 2022–drawing a salary, shares and stock options. RT is an MR sequence developer employed by Hyperfine Inc., and is a holder of shares and stock option of Hyperfine Inc. JOM has institutional funding from the Bill & Melinda Gates Foundation Consortium grant to support research work by his group in neurodevelopment, including data from conventional 3T MRI and Hyperfine scanners–this grant is focused on image analysis and is not commercially sponsored. SW has received funding from the Bill & Melinda Gates Foundation for attendance and travel to sites for training and knowledge exchange during the development and delivery of this project. TA is supported by the UK MRC for a Translational support fellowship [MR/V036874/1] for personal salary, and funding for 3T MRI scans, the MRC Centre for Neurodevelopmental Disorders, King’s College London [MR/N026063/1] for administrative support, funding, and a Clinician Scientist Fellowship [MR/P008712/1]—for personal salary and funding for 3T MRI scans. TA is supported by the EPSRC UK Network grant, co-investigator [EP/W035154/1]. DE and JVH: The Hyperfine machine (Swoop® MR System) was provided by the Bill and Melinda Gates Foundation as part of the Unity Consortium.

## Appendix A Supplementary data

Online Supplement

UNITY Consortium—PubMed Study Group Indexing
