# Supplementary material for: Development of neonatal-specific sequences for portable ultralow field magnetic resonance brain imaging: a prospective, single-centre, cohort study
Source: eClinicalMedicine. 2023 Oct 10;65:102253. doi: 10.1016/j.eclinm.2023.102253 (PMC10725077; doi:10.1016/j.eclinm.2023.102253)
Supplement: Online Supplement [file mmc1.pdf]

Development of neonatal-specific sequences for portable ultralow field magnetic resonance brain imaging: a prospective, single-centre, cohort study– *online supplement*

Paul Cawley MRCPCH [1,2,3], Francesco Padormo PhD [1,4,5], Daniel Cromb MRCPCH [1,2], Jennifer Almalbis MAN [1,2], Massimo Marenzana PhD [1], Rui Teixeira PhD [5], UNITY Consortium [6], Alena Uus PhD[1], Jonathan O'Muircheartaigh PhD[1,3,7], Steven C.R Williams PhD [6], Serena J. Counsell PhD [1], Tomoki Arichi PhD [1,3,8], Mary A. Rutherford MD [1,3], Joseph V. Hajnal FREng [1], A. David Edwards FMedSci [1,2,3]

1. Centre for the Developing Brain, School of Biomedical Engineering and Imaging Sciences, King's College London, London, SE1 7EH, UK
2. Neonatal Intensive Care Unit, Evelina Children's Hospital London, 6th Floor North Wing, St Thomas' Hospital, Westminster Bridge Road, London, SE1 7EH, UK
3. MRC Centre for Neurodevelopmental Disorders, King's College London, London, SE1 1UL, UK
4. Medical Physics, Guy's & St. Thomas' NHS Foundation Trust, London, UK
5. Hyperfine, Inc., 351 New Whitfield St. Guilford, Connecticut, 06437, USA
6. Centre for Neuroimaging Sciences, De Crespigny Park, King's College London, London SE5 8AF, UK
7. Department of Forensic and Neurodevelopmental Science, Institute of Psychiatry, Psychology and Neuroscience, King's College London, London, UK
8. Paediatric Neurosciences, Evelina London Children's Hospital, Guy's and St Thomas' NHS Foundation Trust, London SE1 7EH, UK

Table of Contents

Recruitment & Eligibility Criteria 1
Participant Demographics 2
Participant Characteristics 2
Maternal Ethnicity of Study Cohort 3
Portable Magnetic Resonance Imaging System - Hyperfine Swoop® 4
T1 Weighted Imaging 5
T2 Weighted Imaging 8
Rigid Registration & Signal Averaging 9
Performance of T2w & T1w 64mT Structural imaging: Neuroanatomy 14
T2 Weighted Imaging of the Posterior Limb of the Internal Capsule 14
T1w Imaging of the Corticospinal Tracts and the Posterior Limb of the Internal Capsule 16
Inversion-Recovery Imaging & 64mT-3T Congruence 16
Signal Averaging, Motion, Inversion Time and PLIC Appearances 19
Multiple Inversion Time - T1 Weighted Strategy 20
Congruous and Non-Identified Pathologies 22
Non-Identified Pathology – Punctate White Matter Lesions, Microcysts & Subependymal Cyst 25
Example Imaging: Motion Artefact 28
Example Imaging: Pituitary & Pituitary Stalk 29
Example Imaging: Developmental Brain Abnormalities 30
UNITY Consortium Members 32
Online Supplement References 33

Recruitment & Eligibility Criteria

Recruitment was achieved through convenience sampling of infants undergoing 3T MR brain imaging for risk or suspicion of brain abnormality, or for research into healthy newborn brain development, at the Evelina London Children's Hospital Newborn Imaging Centre.

The Evelina Newborn Imaging Centre is a state-of-the-art MRI clinical research imaging facility purpose-built within a quaternary Neonatal Intensive Care Unit. The Neonatal unit hosts 46 cots with approximately 1,000 admissions (~250 born <30 weeks gestation) annually and ~10,000 Intensive/High-dependency cot days per year. The unit is a referral centre for neonatal sub-specialist care including cardiology, cardiothoracic surgery, surgery, urology, endocrinology, nephrology, respiratory medicine, gastro-enterology, ophthalmology, metabolic medicine, dermatology, Ear Nose & Throat, plastic surgery, cleft lip & palate services, orthopaedic surgery, and clinical genetics. The co-located delivery suite serves approximately 7,000 births per year, many high-risk due to the provision of tertiary maternity, fetal medicine and fetal cardiology services, as well as specialist adult medical, surgical and intensive care services. The Neonatal Unit is also adjacent to an additional 39 bedded post-natal ward for well babies to establish feeding and remain with their mothers as they convalesce post-delivery.[1] Excluding the catchment areas of other referring tertiary centres, our hospital serves a local inner-city population of approximately 625,000.[2]

Recruitment continued until sequence optimisation had been completed and evaluated over a range of gestational ages, and in the presence and absence of a range of developmental and acquired pathologies. No definitive criteria for 'completion' of sequence optimisation were pre-specified as the absence of prior optimisation work at 64mT prohibited projection of achievable contrast, signal, noise, and resolution. Completion of sequence optimisation was thus pragmatically determined once parameter adjustment achieved tissue contrast of white matter, grey matter and CSF, and higher spatial resolution had been pursued to the point where any further augmentation would have compromised either practicable sequence duration for a sleeping infant and/or compromised sequence performance due to reduced visual Signal to Noise Ratio. Similarly, determination of achieving an adequate range of both gestational maturation and pathologies was pragmatically determined; once our optimised sequences had been tested against preterm and term developmental stages and both developmental and acquired abnormalities of the cortex, white matter, deep grey matter and the posterior-limb of the internal capsule. This pragmatic sample was achieved between 23rd September 2021 to 25th October 2022. Sample size calculations were not used.

Infants of any gestational age with risk or suspicion of brain abnormality were eligible for inclusion if they fulfilled any of the following criteria: 1) Clinical concerns about a neurological abnormality on the basis of antenatal findings, clinical course, or a suspected or diagnosed genetic abnormality, 2) When a suspected brain abnormality had been identified on another imaging modality (such as fetal MRI, cranial ultrasound, or CT scan) where an MR scan was clinically indicated to provide better visualisation of any abnormalities, and/or 3) Clinical evidence of neurological abnormality such as seizures, abnormal muscle tone/movements/sensory responses, or difficulty feeding. Clinicians including neuroradiologists could request follow-up scans for recruited infants based on clinical indication (for example to assess evolution of pathological changes to further guide prognosis or refine diagnosis if clinical signs and symptoms progress) – follow-up scans were eligible for inclusion in this study up-until the age of 12 months.

Inclusion criteria for preterm or term infants without suspected neurological abnormality were: Live infants following premature delivery (23 - 36 weeks gestation) or live infants born at full term gestation (37 - 42 weeks gestation). Preterm infants in this group were eligible for repeat scanning at term equivalent age (≥37 weeks post-menstrual age). Exclusion criteria for infants without suspected neurological abnormality were: 1) Infants with major genetic abnormalities and/or chromosomal syndromes, 2) Term infants with a history of in-utero compromise prior to delivery and/or poor condition at delivery requiring vigorous resuscitation and medical care, or 3) Preterm infants who are extremely unwell and therefore would not tolerate the scanning period, despite full intensive neonatal care. The presence of contra-indication for MRI scan (e.g. MR incompatible implant) was an exclusion criterion for all groups. Both infants without suspected neurological abnormality and infants with clinical indications for scanning were eligible for recruitment from either our neonatal unit or post-natal ward.

Study ethics granted use of chloral hydrate for all infants enrolled for 3T imaging only (i.e. not for 64mT scanning in isolation). We reserved use of oral sedation for clinical indications only, unless parents of infants without suspected neurological abnormality indicated an informed preference for oral sedation. We obtain informed written consent for use of chloral hydrate, regardless of indication.

Participant Demographics

Participant Characteristics

Online supplement table S1 shows participant characteristics for those infants receiving longitudinal scanning, including medical support during scan, co-morbidities and the technical focus of participant's scan (T1 / T2 weighted development).

Participant Characteristic	64mT Longitudinal Scans* *n* = 12 infants
Gestation at birth [Weeks^{+days}]	32^{+2} (28^{+6} – 37^{+5})
Weight at birth [Kg]	1·77 (1·25 – 3·24)
Age at scan [Days]	78 (52 – 113)
PMA at scan [Weeks^{+days}]	44^{+5} (41^{+0} – 48^{+0})
Weight at scan [Kg]	4·27 (3·63 – 5·18)
Sex	
Female (%)	3 (25)
Male (%)	9 (75)
Medical Support during scan	
Mechanical ventilation (%)	0 (0)
CPAP (%)	0 (0)
LFNC (%)	2 (17)
Prostaglandin Infusion (%)	0 (0)
Temperature controlled mattress (%)	1 (8)
Inotropic therapy (%)	0 (0)
Arterial line (%)	0 (0)
Given chloral hydrate sedation† for 3T MR scan (%)	1 (8)
Co-Morbidity	
Born Preterm (%)	8 (67)
Congenital cardiac lesions (%)	2 (17)
Encephalopathy, abnormal movements/ Seizures or Suspected HIE (%)	1 (8)
Suspected Developmental Brain Abnormalities (%)	0 (0)
Suspected Metabolic Disorder (%)	1 (8)
Post-Meningitis (%)	1 (8)
Post-ECMO (%)	1 (8)
Focus of Sequence Development:	
Solely T1 weighted sequence development (%)	6 (50)
Solely T2 weighted sequence development (%)	0 (0)
T1 & T2 weighted sequence development (%)	6 (50)

Online Supplement Table S1. Table of Participant Characteristics for Longitudinal 64mT Scans. Continuous Data are shown as: Median (Interquartile Range). PMA: Post-Menstrual Age; CPAP: Continuous Positive Airway Pressure; LFNC: Low Flow Nasal Cannulae (Oxygen); HIE: Hypoxic Ischaemic Encephalopathy; ECMO: Extra-Corporeal Membrane Oxygenation. *Representing 13 scans in 12 infants - abandoned scans not contributing imaging data are excluded (both failed attempts were successful on second attempt on same day). †No infants without suspected neurological abnormality received chloral hydrate in this cohort.

Maternal Ethnicity of Study Cohort
All mothers are asked to self-declare their ethnic background on maternity booking via a list of pre-specified options on their electronic clinical record. For purposes of monitoring of equity, diversity, and inclusion we share the self-declared ethnic background for the mothers of all included participants in **online supplement table S2**.

Self-selected ethnic group	Number (%)
White: British	29 (33)
White: Irish	1 (1)
White: Other	17 (20)
Mixed: White/ Black Caribbean	0 (0)
Mixed: White/ Black African	1 (1)
Mixed: White/ Asian	0 (0)
Mixed: Other	2 (2)
Asian: Indian	1 (1)
Asian: Pakistani	1 (1)
Asian: Bangladeshi	0 (0)
Asian: Other	3 (3)
Black: Caribbean	1 (1)
Black: African	5 (6)
Black: Other	1 (1)
Chinese	3 (3)
Any Other	4 (5)
Declined / Not stated	18 (21)
Total	87 (100)

Online Supplement Table S2. Self-declared maternal ethnicity of study cohort – selected from one of the prespecified options shown in the order of the above rows

Portable Magnetic Resonance Imaging System - Hyperfine Swoop®

The Hyperfine Swoop® is shown in **online supplement figure S1**. The Swoop® system has an inbuilt FDA approved (US FDA K212456) deep learning image reconstruction pipeline for T1w, T2w, and FLAIR imaging. To remove the risk of deep learning having unmeasurable effects on the imaging appearances of our optimisations, we deactivated the deep-learning output - no machine/deep learning, or artificial intelligence has been used in the reconstruction of imaging used in this work.

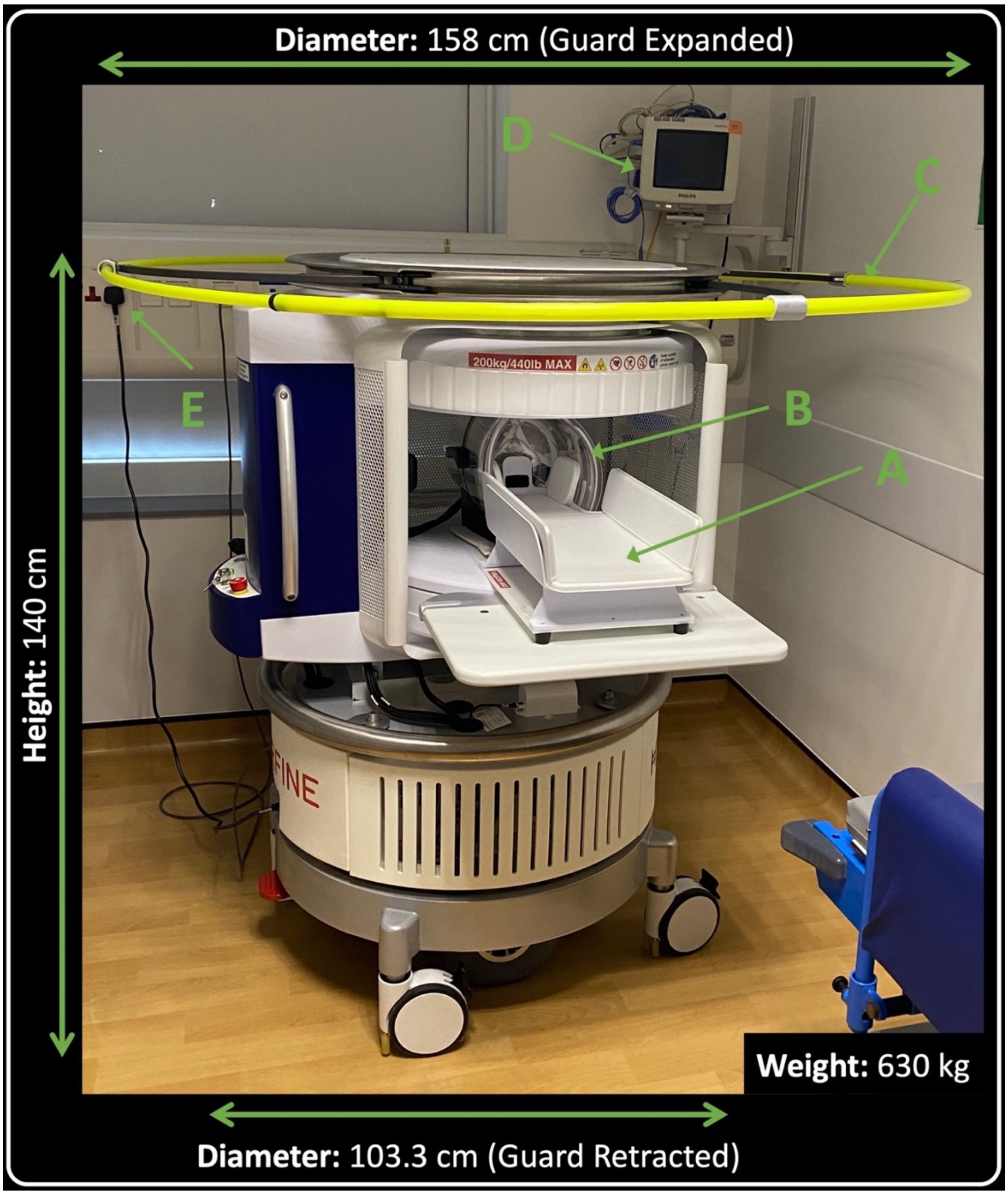

Online Supplement Figure S1. 64mT Hyperfine Swoop® with "Baby Nest" infant cradle (A), and 8 channel head coil (B). Extended yellow halo (C) demarcates the 5 Gauss MR safety perimeter. The scanner is in proximity of a standard bedside monitor (D) and is powered by a standard mains electrical socket (E).

Manufacturer standard sequences shown in this manuscript are solely from software version 8.2.0; manufacturer standard sequences from subsequent software updates have not been tested in this work.

The Hyperfine swoop has built-in radiofrequency interference rejection methods. Pre-scan calibration and auto-align protocols, each lasting 15 to 31 seconds, are performed at the start of scanning sessions and 2 second 'fine' calibrations performed prior to initiation of each sequence.

Technical Development of 64mT Neonatal Sequences

Low resolution Inversion-Recovery T1 Mapping was undertaken to guide sequence development[3] and T1 & T2 weighted 64mT sequence parameters were iteratively changed to study the impact of resolution, bandwidth, field of view, time to echo, repetition time and inversion time. Successful alterations were used as a basis for further optimisations.

T1 Weighted Imaging

Parameters for each T1 weighted sequence generation are shown in **online supplement table S3**. Examples of each T1 weighted sequence generation are provided in **online supplement figure S2,** and a further comparison of T1w Manufacturer Standard Adult 64mT sequence, reference 3T imaging and dedicated 64mT neonatal sequence is shown in **online supplement figure S3.**

Sequence Generation	Scan Plane	Resolution (mm)	Field of View (mm)	Bandwidth (kHz)	TE (ms)	TR (ms)	TI (ms)†	Turbo Factor	Duration (Min Sec)
**0 - Manufacturer Standard (Adult)**	Axial	1·6 x 1·6 x 5·0	220 x 180 x 180	64	5·96	1,500	300	24	5min 38s
**1 - First**	Axial	1·6 x 1·6 x 5·0	220 x 180 x 180	64	5·96	1,500	200, 400, 500, 600, 700, 800	24	5min 38s
**2 - Second***	Axial	2·0 x 2·0 x 2·0	180 x 240 x 240	32	7·16	1,500	600	48	9min 59s
**3 - Third (A)**	Axial	2·0 x 2·0 x 2·0	180 x 240 x 240	32	7·16	840	Non-Inversion	48	5min 36s
**3 - Third (B)**	Axial	2·0 x 2·0 x 2·0	180 x 240 x 240	32	7·16	2860	Non-Inversion	48	19min 0s
**3 - Third (C)**	Axial	2·0 x 2·0 x 2·0	180 x 240 x 240	32	7·16	1500	750, 800, 850	48	9min 59s
**Low Resolution***	Axial	2·8 x 2·8 x 2·8	180 x 220 x 180	64	4·62	1,500	100, 200, 300, 400, 500, 600, 700, 800, 900, 1010 & non-inversion	48	3min 33s
**Low Resolution**	Axial	2·8 x 2·8 x 2·8	180 x 220 x 180	64	4·62	3,000	900	48	7min 04s
**4 - Fourth***	Axial	2·0 x 2·0 x 2·0	180 x 220 x 180	32	7·16	1,500	300, 350, 400, 450, 500	48	6min 55s

Online Supplement Table S3. T1 Weighted Sequence Generations: Time to Echo (TE), Repetition Time (TR), Inversion Time (TI). Resolution and Field of View listed in order of scan plane. *Averaging trialled during this sequence generation. †Multiple inversion times indicates multiple TIs trialled during that generation of scan. *Nb. Transient/experimental non-optimised sequences with minimal use not shown for brevity (4 sequences in 2 infants with other 64mT imaging).*

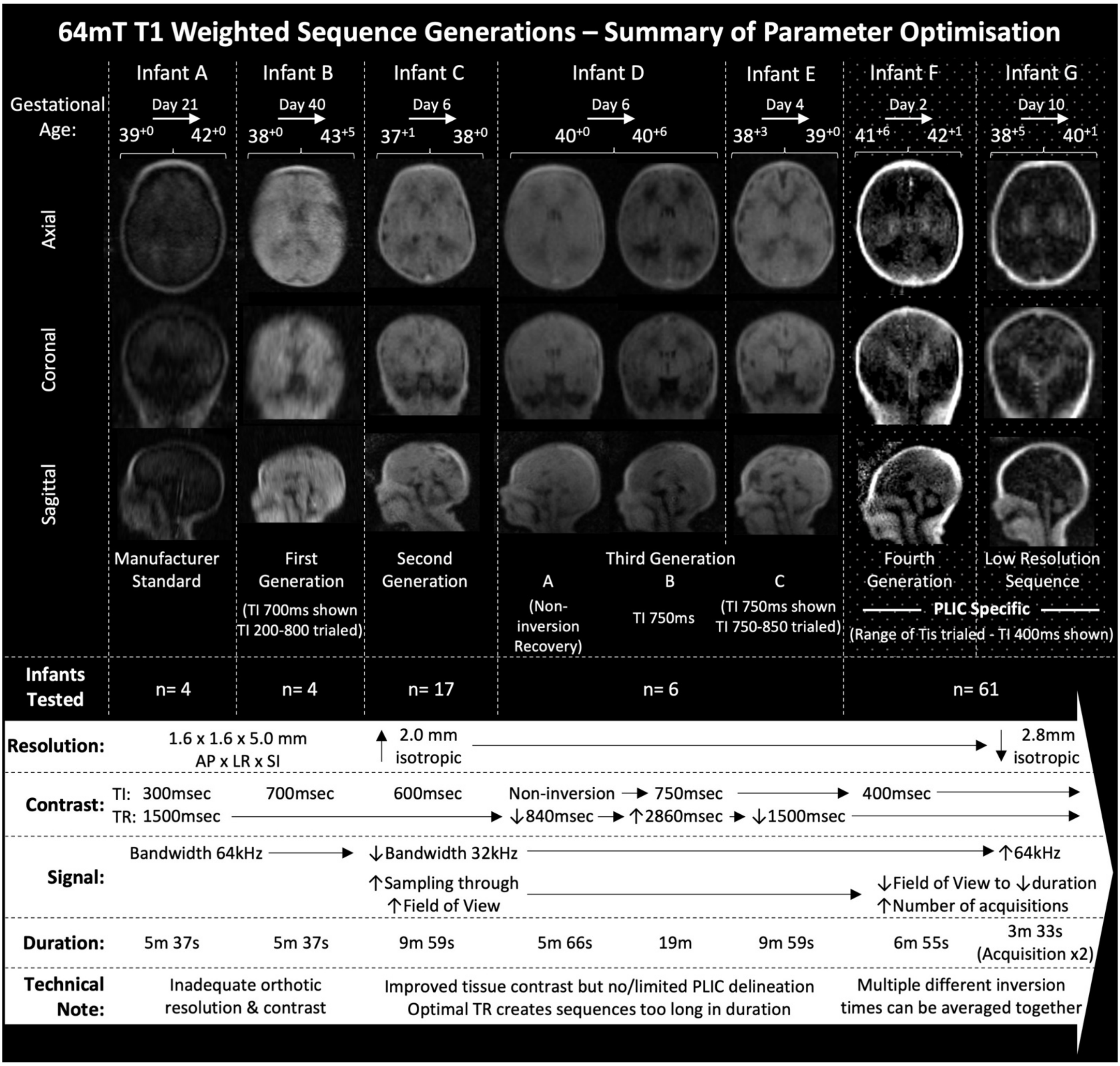

Online Supplement Figure S2. T1 Weighted Sequence Generation – Summary of Parameter Optimisations. Example images from each sequence generation, alongside iterative optimisation of parameters to enhance resolution, signal, and contrast for T1w imaging.

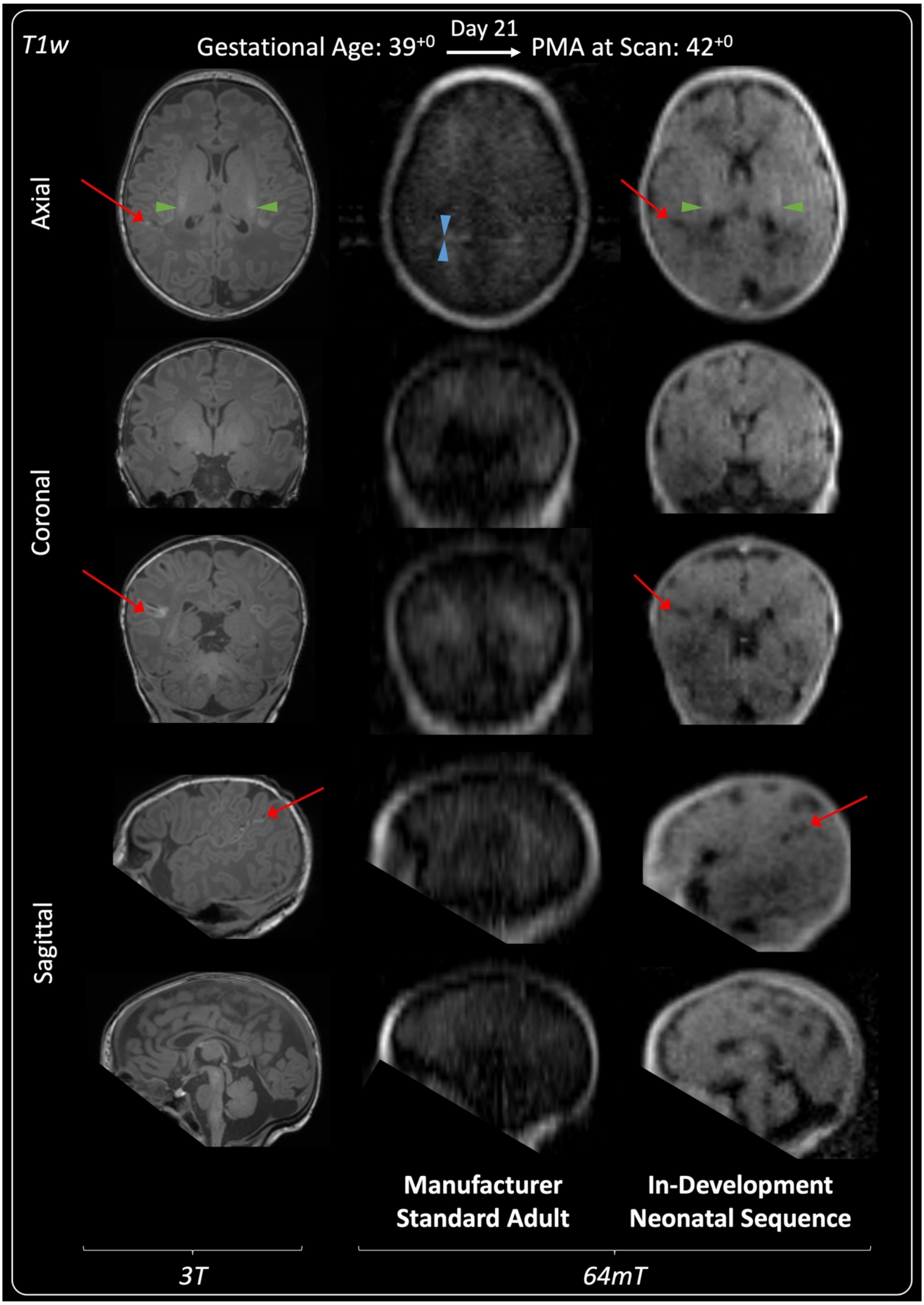

Online Supplement Figure S3. T1 weighted Manufacturer Standard Adult 64mT sequence compared with reference 3T imaging and dedicated 64mT neonatal sequence. Term infant with small right-sided focal cortical infarction in the superior parietal lobe (red arrows). 3T MR scan was performed on day 15 and 64mT scan on day 21. T1w imaging: 64mT Adult T1w imaging sequence is unable to differentiate the cortex, white matter, deep grey matter, or ventricles; normal high T1 signal in the Posterior Limb of the Internal Capsule (PLIC - single green arrowheads) is not visible. A zipper artefact is seen (blue double arrowheads). Neonatal 64mT sequence allows PLIC T1 signal, and subtle cortical T1 hypointensity at the site of the infarction to be visualised.

T2 Weighted Imaging
Parameters for each T2 weighted sequence generation are shown in **online supplement table S4**. Examples of each T2 weighted sequence generation are provided in **online supplement figure S4.**

Sequence Generation	Scan Plane	Resolution (mm)	Field of View (mm)	Bandwidth (kHz)	TE (ms)	TR (ms)	Turbo Factor	Duration (Min Sec)
**0 - Manufacturer Standard Adult**	Axial	1·5 x 1·5 x 5·0	180 x 220 x 180	64	204	2000	80	5min 57s
**0 - Manufacturer Non-Standard (Neonatal)**	Axial	1·5 x 1·5 x 5·0	220 x 180 x 180	64	260	2000	80	5min 50s
**1 - First**	Axial	2·25 x 2·25 x 2·25	220 x 180 x 180	64	266	2000	104	6min 40s
**2 - Second**	Axial	2·25 x 2·25 x 2·25	240 x 220 x 240	64	273	2000	104	5min 26s
**3 - Third**	Axial	2·25 x 2·25 x 2·25	180 x 220 x 180	32	269	2000	84	9min 58s
**4 - Fourth**	Axial	2·25 x 2·25 x 2·25	180 x 220 x 180	32	269	2000	84	5min 4s
**5 - Fifth***	Axial	2·0 x 2·0 x 2·0	180 x 240 x240	32	291	2000	84	9min 10s
**6 - Sixth***	Axial	1·75 x 1·75 x 1·75	182 x 238 x 238	32	290	2000	76	12min 56s
**7 - Seventh***	Sagittal	2·25 x 2·25 x 2·25	224 x 123 x 140	32	281	2000	66	4min 38s

Online Supplement Table S4. T2 Weighted Sequence Generations: Time to Echo (TE), Repetition Time (TR). Resolution and Field of View listed in order of scan plane. *Averaging trialled during this sequence generation. *Nb. Transient/experimental non-optimised sequences with minimal use not shown for brevity (2 sequences in 3 infants with other 64mT imaging).*

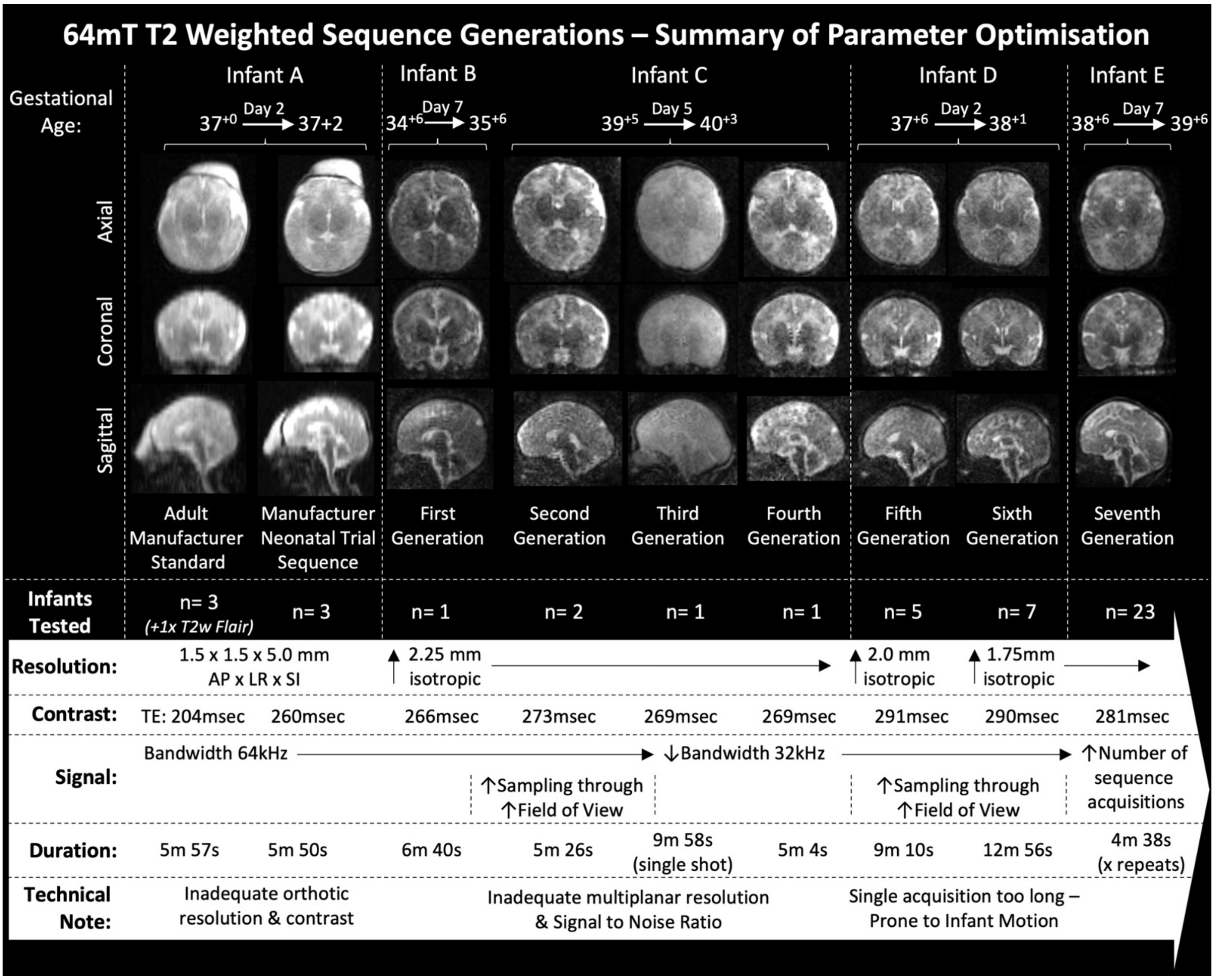

Online Supplement Figure S4. T2 Weighted Sequence Generation – Summary of Parameter Optimisations Example images from each sequence generation, alongside iterative optimisation of parameters to enhance resolution, signal, and contrast for T2w imaging

Rigid Registration & Signal Averaging

Sequence alternations which enhanced resolution or were made to increase signal-to-noise ratio (SNR) led to increased sequence durations, increasing the risk of infants waking or moving during any one sequence. Once parameters had been identified to provide adequate tissue contrast, we switched to performing multiple short sequences which could then be registered and averaged to enhance SNR – as shown in **online supplement figure S5**, discarding single sequences distorted by motion. The process of running each scan through the relevant script for registration and averaging (FSL and FLIRT) takes approximately 2 to 5 minutes. This process would be impractical in the clinical context and requires automation prior to introduction into the clinical environment. We are now working directly with the manufacturer to automate this function within the Swoop® system.

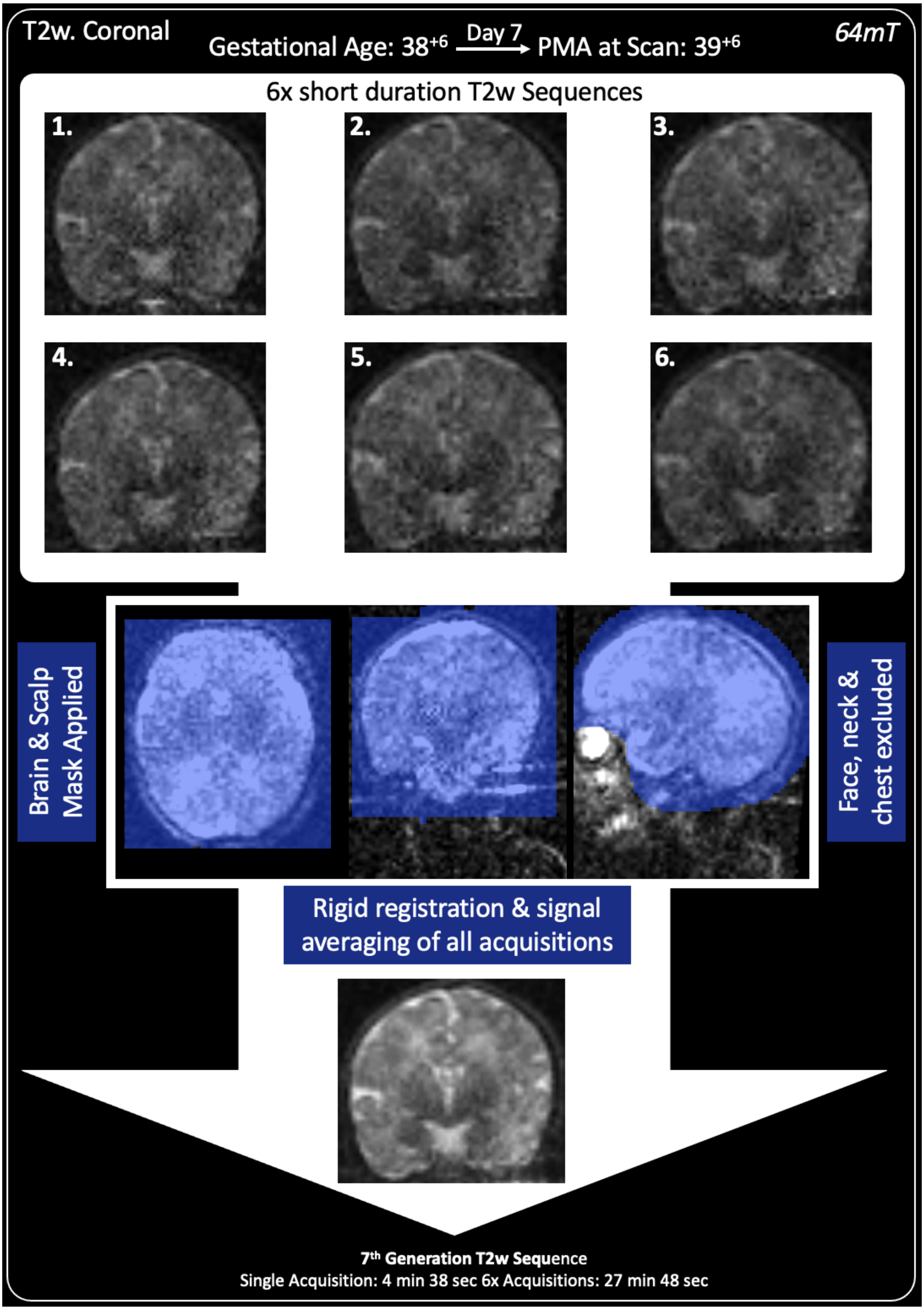

Online Supplement Figure S5. Rigid registration and averaging: 6 repeats of the same T2 weighted Fast Spin Echo Sequence are performed. Individually these are low signal / high noise. A brain and scalp mask is applied to all acquisitions and rigid registration performed to account for inter-acquisition movement. Once registered, signal can be averaged to produce the final high signal / low noise image at the bottom of the figure.

Side-by-side comparison of Manufacturer Standard Adult 64mT sequences and optimised neonatal 64mT sequences, with use of signal averaging for both T1w and T2w imaging, is provided in **online supplement figure S6**.

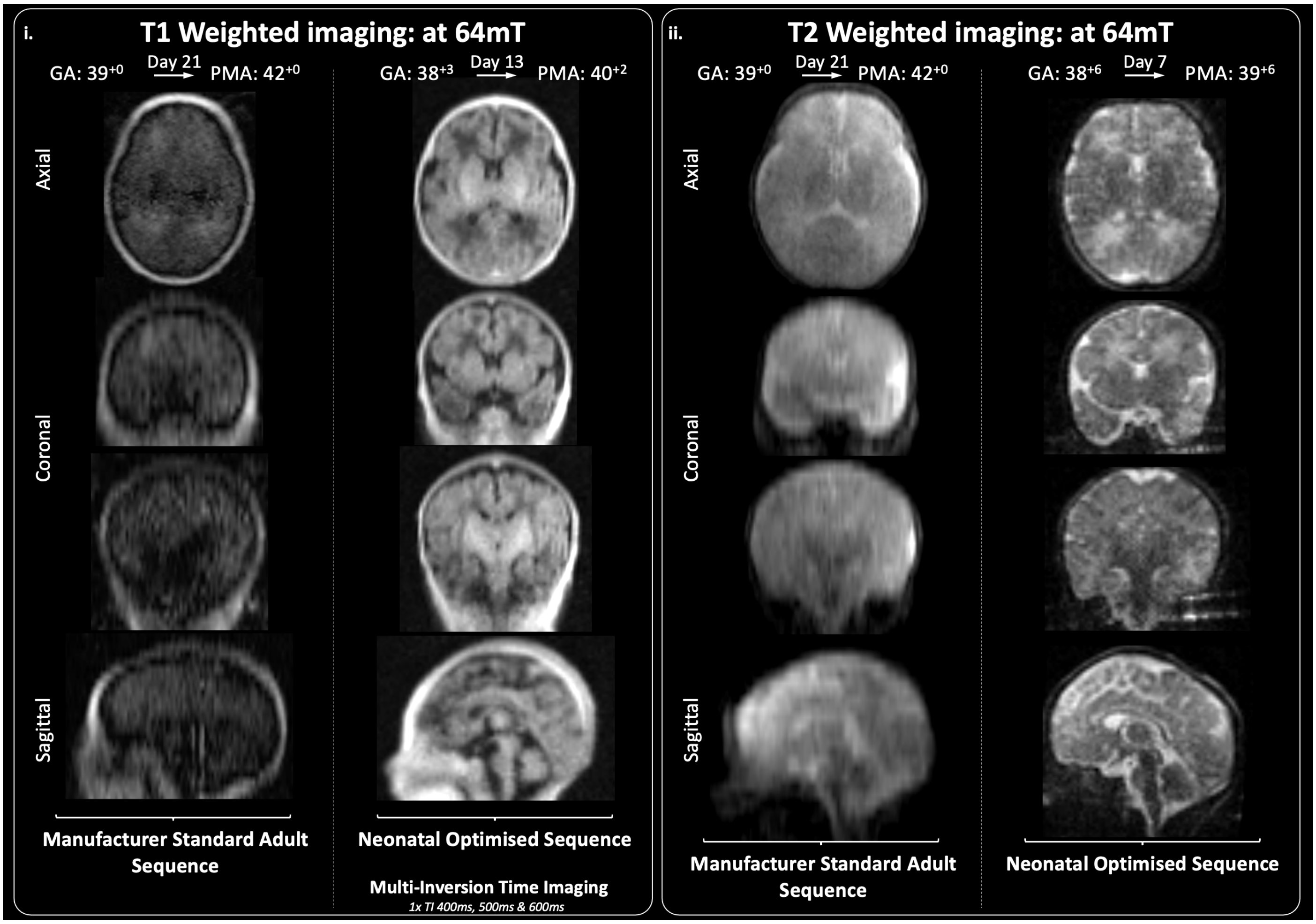

Online Supplement Figure S6. Side-by-side comparison of Manufacturer Standard Adult Sequences and neonatal optimised sequences at 64mT for i. T1 weighted Imaging and ii. T2 weighted Imaging. Gestational Age (GA), Post-Menstrual Age (PMA).

The total scan time of a sequence is a direct multiple of the number of acquisitions performed – Each repeat acquisition thus enhances the SNR at the expense of increased scanning time. A balance between SNR to scan duration must therefore be found and this may vary according to the application the scan is being used for (e.g. research vs clinical and what research or clinical questions are being asked). **Online supplement figure S7** shows scan appearance by acquisition number (one to six). Visual distinction of slight tissues and anatomical structures (e.g. cortex, optic nerves, pituitary stalk/tissue, corpus callosum) is more challenging when averaging with fewer acquisitions is used (e.g. one to three acquisitions); so clinical or research questions requiring better resolution of the cortex and these anatomical structures may require longer scanning protocols to resolve SNR, than for other basic screening applications.

Impact on visual performances of 1-3 versus ≥5 acquisitions for signal averaging of our T2w sequence generation 7 are shown in **online supplement figure S8 – for all infants, excluding follow-up scans**. Percentage visualisation is higher in the cohort with the greater number of acquisitions for all anatomic structures and tissues, with the exception of visualisation of the course of the vestibulocochlear nerve (1-3 acquisitions – 44% vs ≥5 acquisitions – 14%) and visualisation of the inner ear (1-3 acquisitions – 69% vs ≥5 acquisitions – 43%). Cause of failure in the ≥5 acquisition cohort was obfuscation of the vestibulocochlear nerve and inner ear structures by the zipper artefact in 4 infants (57%), with signal averaging augmenting the zipper artefact. Zipper artefact is improved on later manufacturer software advances through enhanced interference removal. None of the ≥5 acquisition group were imaged using the later manufacturer software with enhanced interference removal, whereas 5 of the 1-3 acquisition group were imaged using the new interference removing software; in this group of 5 the inner ear structures were visualised in 5/5 infants (100%) and the vestibulocochlear nerves in 3/5 infants (60%). Furthermore, an additional 3 infant scans can be added to these 5, to create a sub-cohort of 8 infants – this including infants with follow-up scans using the new software which were excluded from the original analysis; their first scans performed on the old software version. In this 8 infant cohort using optimised 64mT T2w scans (all with only 1 or 2 total acquisitions for averaging), and the new software, the vestibulocochlear nerve was visualised in 5/8 infants (63%) and the inner structures visualised in 7/8 infants (88%).

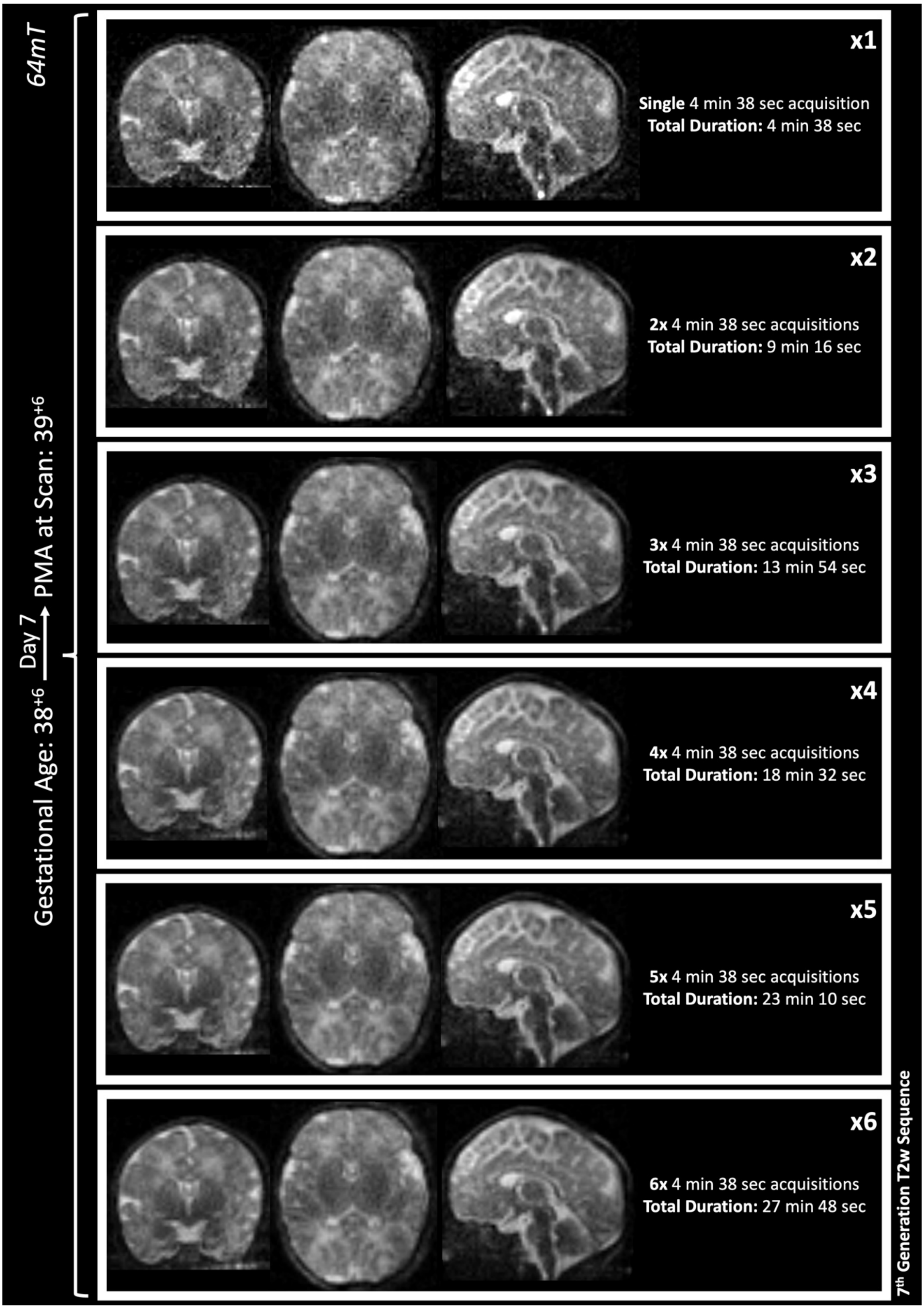

Online Supplement Figure S7. Visual appearance of T2w imaging by number of acquisitions included in sequence averaging. Post-Menstrual Age (PMA).

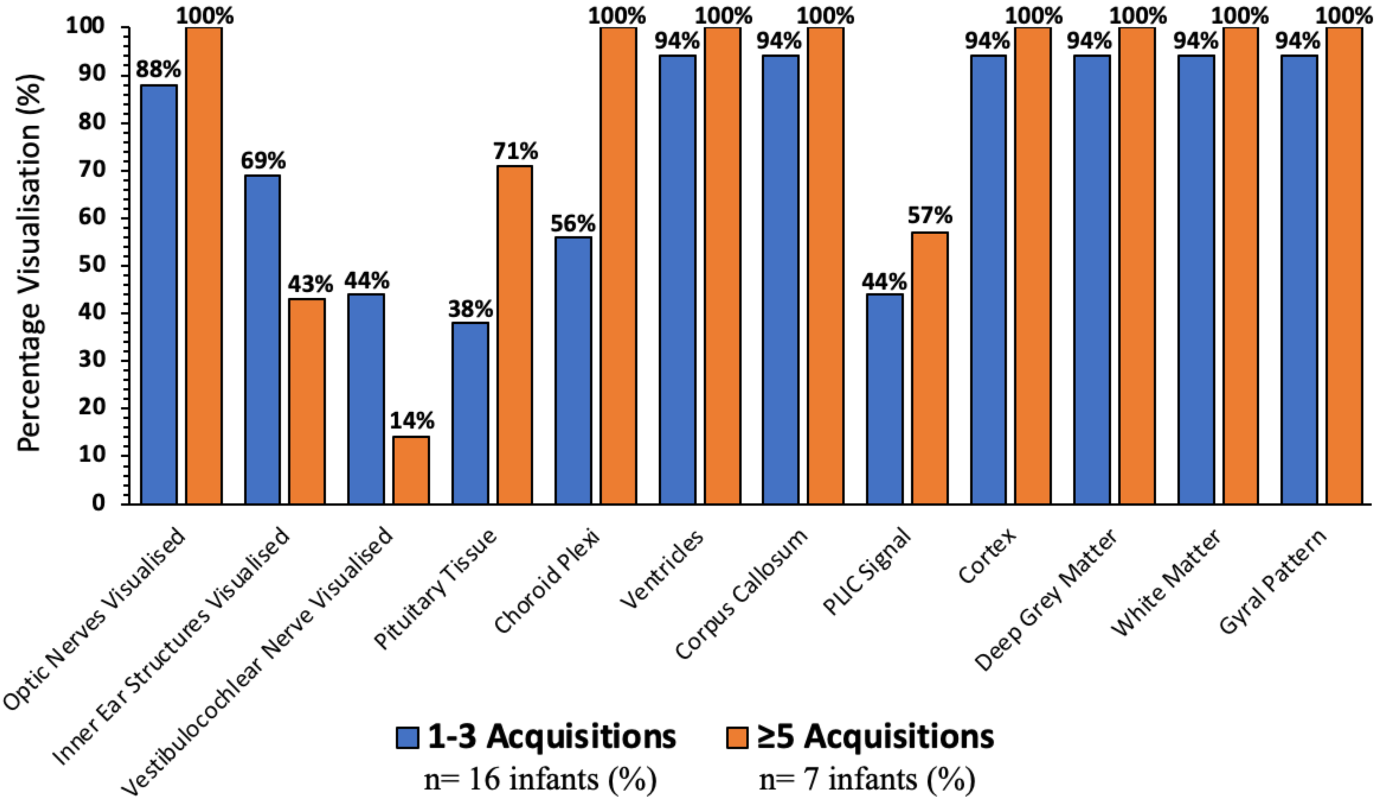

Online Supplement Figure S8. Percentage visualisation of anatomical structures/tissues by number of 64mT T2w acquisitions included in sequence averaging: 1-3 Acquisitions versus ≥5 Acquisitions – Sequence Generation 7 only (No infants underwent solely 4 acquisitions). Signal averaging augmented historical zipper artefact and contributed to the diminished performance of visualisation of the vestibulocochlear nerve and inner ear structures. Zipper artefact is improved on later manufacturer software advances.

Performance of T2w & T1w 64mT Structural imaging: Neuroanatomy

The neuroanatomical and brain tissue visual performances of 64mT T2w sequence generations 0–4 and 5–7, and 64mT T1w sequence generations 0–1 and 2–3 are further summarised in **online supplement table S5**.

		T2w Neonatal Development & Manufacturer Standard Sequences (Generations 0-4) n= 10 infants (%)	T2w Neonatal Optimised Sequences (Generations 5-7*) n= 34 infants (%)	T1w Neonatal Development & Manufacturer Standard Sequences (Generations 0-1) n= 7 infants (%)	T1w Neonatal Optimised Sequences (Generations 2-3) n= 23 infants (%)
Structures Visualised	Optic Nerves	1/10 (10)	31/34 (91)	0/7 (0)	19/23 (83)
	Inner Ear Structures	4/10 (40)	22/34 (65)	0/7 (0)	0/23 (0)
	Vestibulocochlear Nerve	2/10 (20)	9/34 (26)	0/7 (0)	0/23 (0)
	Pituitary	1/10 (10)	20/34 (59)	0/7 (0)	10/23 (43)
	Choroid plexi	3/10 (30)	27/34 (79)	0/7 (0)	18/23 (78)
	Ventricles	8/10 (80)	33/34 (97)	0/7 (0)	23/23 (100)
	Corpus Callosum	1/10 (10)	33/34 (97)	0/5 (0)	9/23 (39)
Tissues Visualised	PLIC Myelin Signal	1/10 (10)	17/34 (50)	1/7 (14)	9/23 (39)
	Cortex	2/10 (20)	32/34 (94)	2/7 (29)	17/23 (74)
	Deep Grey Matter	7/10 (70)	32/34 (94)	2/7 (29)	16/23 (70)
	White Matter	3/10 (30)	33/34 (97)	2/7 (29)	16/23 (70)
	Gyri	1/10 (10)	31/34 (91)	0/7 (0)	4/23 (17)

Online Supplement Table S5. 64mT T2w & T1w visual tissue/structural performance for manufacturer standard / in-development sequences versus optimised sequences. More than one sequence generation may be performed on an individual infant. *Generation 7 sequences are averaged when repeat acquisitions are available – median number of acquisitions = 2 (Interquartile Range 1-5). If serial scans available for any individual infant, the first scan is used for analysis. Posterior Limb of the Internal Capsule (PLIC).

T2 Weighted Imaging of the Posterior Limb of the Internal Capsule

As shown within the main manuscript – **figures 3.i, 5.ii & 6.ii**, the PLIC on T2w imaging is identifiable as a T2 hyperintense streak, bisecting through the central deep grey matter structures. The high T2 signal PLIC, in the absence of myelin, was more prominent within **figure 3.i & 5.ii** due to prematurity, and in **figure 6.ii** due to hypoxia-ischaemia leading to abnormal high T2 signal in the PLIC and abnormally low but contrasting T2 signal in the adjacent ventrolateral nuclei of the thalami and lentiform nuclei. Myelin-like low T2 signal within the PLIC at term age is difficult to distinguish from the generalised and relatively T2 hypointense deep grey matter structures on 64mT images. Thus, instances of PLIC congruence on T2w 64mT sequences were frequently related to the absence of low T2 myelin in preterm states. **Online supplement figure S9** demonstrates a series of PLIC congruent T2w images – however, from this series it is apparent that distinguishing the PLIC on T2w 64mT images can be challenging due to the presence of obfuscating noise and areas of low T2 signal within the basal ganglia outside of, but adjacent to, the PLIC (e.g. particularly the ventrolateral nucleus of the thalamus, as shown in the figure). Myelin-like signal is better assessed on T1w imaging at higher field strengths – this may be the case at ULF and is explored in the next section below.

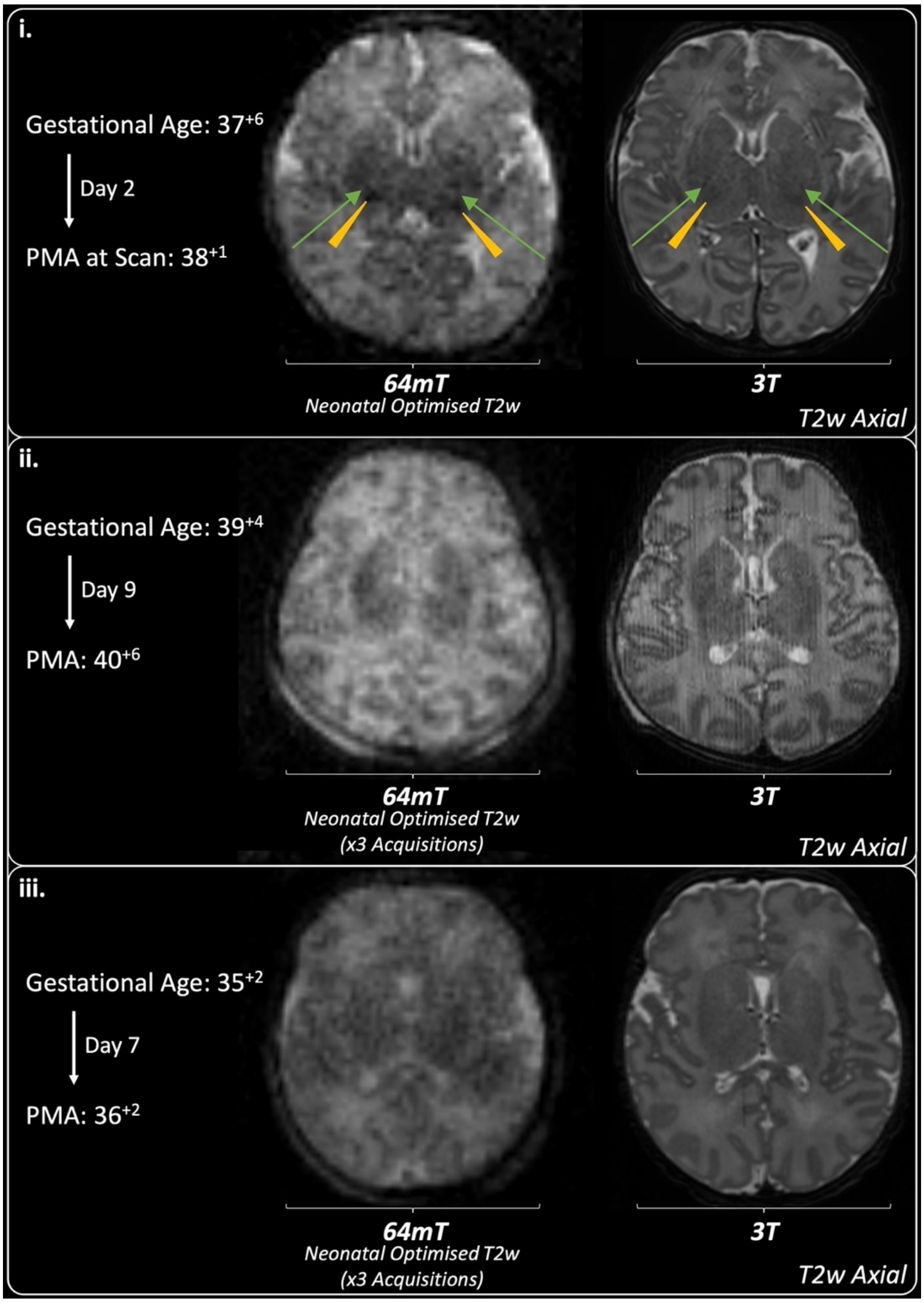

Online Supplement Figure S9: T2w PLIC Appearances. Series of three infants with congruence of PLIC signal: i. Term infant – high T2 signal tract of the PLIC is faint and difficult to distinguish on both 64mT and 3T imaging, small area of low T2 signal within this tract is identified by the green arrows (clearer on infant's right-side). Posterior to these are the low T2 signal ventrolateral thalamic nuclei which appear relatively more hypointense at 64mT. ii. Term infant with absence of myelin signal due to perinatal hypoxia-ischaemia. iii. Infant with maturity-appropriate absence of myelin signal due to prematurity.

T1w Imaging of the Corticospinal Tracts and the Posterior Limb of the Internal Capsule

Inversion-Recovery Imaging & 64mT-3T Congruence
Imaging the Posterior Limb of the Internal Capsule (PLIC) is of major importance in the management of infants with suspected hypoxic-ischaemic encephalopathy, and in the assessment of motor prognosis in all infants imaged at term-equivalent gestations.

We observed putative visual congruence in myelin signal between 3T and 64mT images within only 1 of the 21 (5%) Manufacturer Standard & first generation neonatal T1w sequences in 7 infants and only 10 of 30 (33%) of our optimised T1w structural sequences in 23 infants (T1w Generations 2 &3). We undertook development of 64mT PLIC-specific imaging, with investigation of both low- and high-resolution sequences, in a total of 61 infants, over a range of gestations, and a range of PLIC appearances on 3T.

Consistently, the visual presence and absence of myelin-like PLIC signal against deep-grey matter structures was most appreciable at an inversion time (TI) of 400ms (Repetition time consistent at 1500ms) – The results of our low-resolution 2.8mm isotropic imaging (undertaken to allow a large number of differing inversion times within one scanning session) are summarised in **online supplement table S6**. Low resolution sequences at TI 400ms and TI 500ms showing the highest performance for the presence of any high T1 signal within the expected area of the PLIC – taken as putative myelin T1 signal in 20/22 (91%) and 18/25 (72%) infants respectively in a sub-cohort of infants with confirmed myelin signal on 3T imaging. It should be noted that a number of these 'putative congruent' results at low resolution may be due to visible high T1 noise which is not evident at other inversion times when deep grey matter signal is relatively higher signal. Ability to identify the tract of the PLIC dissecting through the deep grey matter was limited at 64mT (highest again at TI400ms and TI500ms– both 3%) – suggesting that an isointense deep grey matter is congruent with absence of T1 myelin signal on 3T – even if a hypointense PLIC tract is not visualised, when imaged at the correct inversion time.

Imaging at higher resolution, 2mm isotropic, demonstrated a similar T1 contrast profile of the myelinated PLIC, unmyelinated PLIC and the deep grey matter (**online supplement figure S10**). Visual PLIC congruence is shown in **online supplement table S7**. We found it easier to discern putative PLIC signal from potential high T1 noise at the higher resolution. Whilst TI400ms appears to have a lesser 3T PLIC congruence than TI350ms, and a similar congruence profile to TI450ms, the 400ms sequence has been tested more extensively in infants with more subtle PLIC appearances on 3T imaging, as demonstrated by the wider gestational range.

Low Resolution T1w Sequences	TI	Tract of the PLIC visualised (i.e. dissection through the DGM on axial plane) on 64mT imaging<br><br>n= number of infants (%)	Putative PLIC myelin signal present on 64mT imaging in infants with signal confirmed on 3T imaging<br><br>n= number of infants (%)	Potential use for investigation of the PLIC & myelin signal
TI as stated, TR 1500ms<br><br>3min 33s sequence – single acquisitions	TI 100ms	0/29 (0)	0/22 (0)	Unable to contrast either T1 hypointense PLIC tract nor T1 hyperintense myelin signal from signal of deep grey matter
	TI 200ms	0/12 (0)	0/11 (0)	Unable to contrast T1 hyperintense myelin signal from signal of deep grey matter<br><br>Possible selective nulling of the ventrolateral thalami observed (could be used in future applications to anatomically delineate the posterior border of the PLIC but would need further investigation & technical workup)
	TI 300ms	0/30 (0)	3/23 (13)	Putative T1 myelin signal inconsistently identified against nulled DGM, PLIC tract not visible
	TI 400ms	1/29 (3)	20/22 (91)	Putative high T1 signal within expected location of PLIC contrasted against relatively low signal DGM. T1 hypointense tract of PLIC only seen in setting of abnormally high T1 DGM. Due to nulling of non-myelinated white matter and grey matter, only consistently useful anatomical information provided by scalp-fat
	TI 500ms	1/35 (3)	18/25 (72)	Putative high T1 signal within expected location of PLIC contrasted against DGM, contrast visually less than at TI 400ms as DGM have relatively higher T1 signal. T1 hypointense tract of PLIC only seen in setting of abnormal DGM. Location of PLIC easier to identify as other anatomical landmarks within brain are not nulled
	TI 600ms	0/29 (0)	5/22 (23)	Unable to contrast T1 hypointense PLIC tract. DGM have higher T1 signal vs TI 400ms & 500ms leading to inconsistent contrast of putative myelin signal from deep grey matter
	TI 700ms	0/11 (0)	0/10 (0)	Unable to contrast either T1 hypointense PLIC tract nor T1 hyperintense myelin signal from signal of deep grey matter
	TI 800ms	0/28 (0)	0/21 (0)	Unable to contrast either T1 hypointense PLIC tract nor T1 hyperintense myelin signal from signal of deep grey matter
	TI 1010ms	0/19 (0)	0/13(0)	Unable to contrast either T1 hypointense PLIC tract nor T1 hyperintense myelin signal from signal of deep grey matter
	Non-inversion	0/9 (0)	0/8 (0)	Unable to contrast either T1 hypointense PLIC tract nor T1 hyperintense myelin signal from signal of deep grey matter
TI as stated<br><br>7min 4s sequence – single acquisition	TI 900ms TR3000	0/16 (0)	3/10 (30)	Similar profile to TR1500ms & TI 600ms, with slightly higher T1 signal in DGM – likely masking T1 signal of PLIC

Online Supplement Table S6. 64mT–3T visual PLIC tract and signal congruence of low-resolution T1w generation sequences. If serial scans available for any individual infant using the same sequence, the first scan is used for analysis. Posterior Limb of the Internal Capsule (PLIC), Inversion Time (TI), Repetition Time (TR).

		PLIC Signal Congruent with 3T<br><br>n= number of infants (%)	PMA at scan<br><br>Median (Range)
**4th Generation PLIC Specific T1w Sequences**<br><br>**6min 55s – single acquisitions**	TI 300ms Only	0/5 (0)	39^{+3} (36^{+2} – 42^{+1})
	TI 350ms Only	2/2 (100)	41^{+2} (40^{+3} – 42^{+1})
	TI 400ms Only	16/22 (73)	39^{+6} (34^{+4} – 46^{+5})
	TI 450ms Only	5/8 (63)	39^{+6} (35^{+3} – 46^{+5})
	TI 500ms Only	12/19 (63)	39^{+3} (34^{+4} – 46^{+5})
**2nd Generation T1w Sequence**<br><br>**9min 59s – single acquisitions**	**2nd Generation (TI 600ms)**<br>**9min 59s – single acquisitions**	7/17 (41)	40^{+2} (31^{+3} – 53^{+4})

Online Supplement Table S7. 64mT-3T visual PLIC signal congruence of the 2nd and 4th Generation sequences. If serial scans available for any individual infant using the same sequence, the first scan is used for analysis. Posterior Limb of the Internal Capsule (PLIC), Post-Menstrual Age (PMA), Inversion Time (TI).

Signal Averaging, Motion, Inversion Time and PLIC Appearances

Similar to the signal averaging employed in our 64mT T2w imaging, we found that signal averaging of our PLIC-specific T1w sequences (TIs 300, 350, 400, 450, 500, & 600) increased PLIC signal congruence due to enhanced visual SNR. These results are summarised in **online supplement figures 10 and 11**, and **online supplement table S8.**

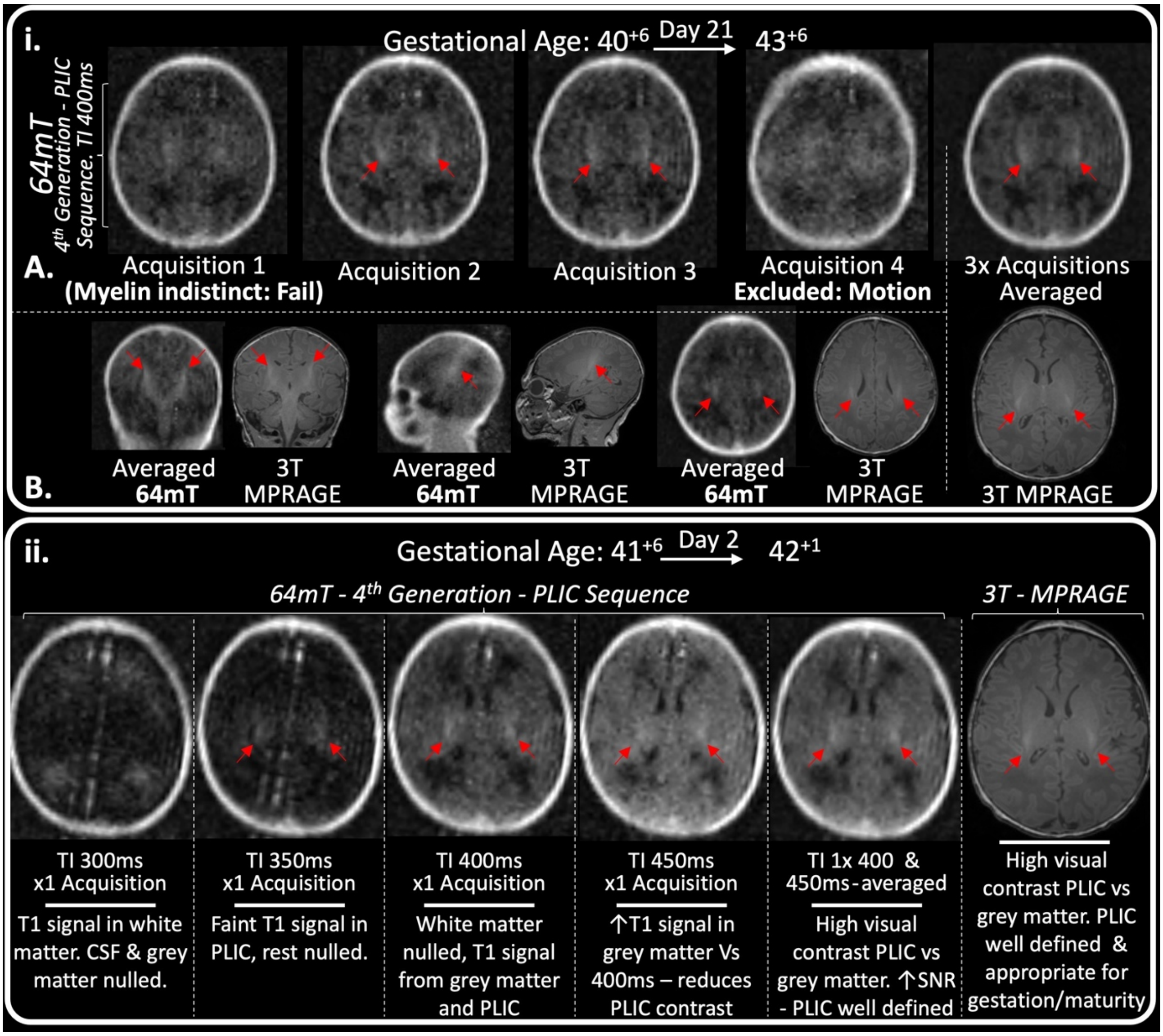

Online Supplement Figure S10: Signal Averaging, Motion, Inversion Time and PLIC Appearances. 64mT T1w bespoke PLIC sequences. Red arrows indicate normal T1 PLIC/corticospinal tract signal. i. A) High T1 signal generated by myelination within the Posterior Limb of the Internal Capsule on axial slices at the level of the thalamus/basal ganglia. Sequence averaging enhances visual SNR and contrast-noise ratio. B) T1 signal generated by myelination within the superior corticospinal tracts, demonstrated in coronal, sagittal and superior axial slices. ii. Differential tissue nulling according to Inversion Time (TI) in a single infant.

Low Resolution PLIC-Specific T1w Sequences with averaging<br><br>3min 33s sequence: averaged 2-8 times	PLIC Signal Congruent with 3T<br><br>n= number of infants (%)	PMA at scan<br><br>Median (Range)
TI 300ms Only	0/1 (0)	Term
TI 400ms Only	6/6 (100)	40^{+4} (37^{+5} – 45^{+5})
TI 500ms Only	5/5 (100)	40^{+1} (39^{+2} – 42^{+1})

Online Supplement Table S8. 64mT–3T visual PLIC signal congruence for low-resolution (2.8mm isotropic) PLIC-specific T1w sequences with at least one additional sequence repetition for signal averaging. If serial scans available for any individual infant the first scan is used for analysis. Posterior Limb of the Internal Capsule (PLIC), Post-Menstrual Age (PMA), Inversion Time (TI)

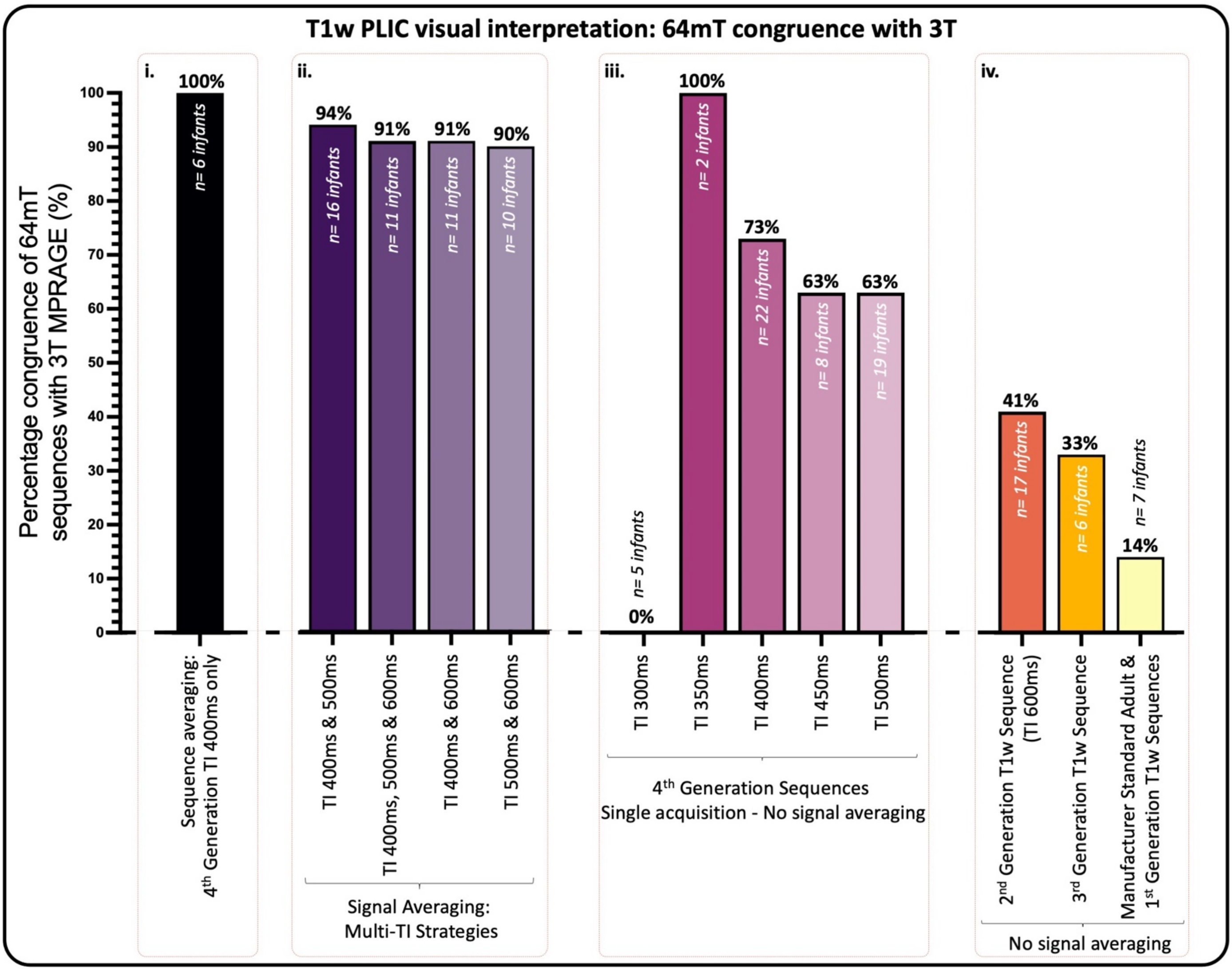

Online Supplement Figure S11. Column chart demonstrating percentage 64mT-3T PLIC signal congruence for high-resolution (2mm isotropic) sequences. i) 4th generation sequence at 400ms with signal averaging; ii) signal averaging of 4th Generation multi-inversion strategies; iii) 4th generation sequences by inversion time, no signal averaging; and iv) earlier generation sequences. If serial scans or sequences available for any individual infant, the first scan is used for analysis. TI: Inversion Time.

Multiple Inversion Time - T1 Weighted Strategy

64mT PLIC-Specific inversion times (principle example TI 400ms) achieve congruence with 3T imaging through both augmentation of the neonatal PLIC signal and attenuation of the surrounding tissues (Deep Grey Matter), thus creating contrast. Signal within neonatal brain tissues other than the PLIC are, however, also attenuated or nulled at these same inversion times – meaning these sequences perform poorly at identification of other structures and are therefore not sufficient for stand-alone T1w structural imaging. The visual structural performance of the 400ms PLIC-specific sequence is shown in **online supplement table**

S9. Other sequences which have shown better overall structural performance (e.g. T1w generations 2 and 3) of other brain tissues have performed poorly at the identification of PLIC myelin signal.

Our investigations have led us to conclude that attempts to create one specific short duration T1 weighted sequence able to achieve contrast between all major MR brain tissues at the same time – white matter, grey matter, CSF and, importantly, myelin like signal within the PLIC, are unlikely to be successful. Instead, a multiple inversion strategy may prove to be more successful, with multiple sequences of varying, but specifically selected, TIs used to augment and null select brain tissues. Post-acquisition processing can then combine these sequences to produce images with sufficient contrast between all tissues, as well as benefit from the increased SNR achieved from the multiple acquisitions.

One such strategy is demonstrated within **figure 4.ii** in the main article (and **online figure S6** in this supplement), superimposition of PLIC signal onto our optimised T1w structural imaging was possible and could further enhance the visual SNR of the PLIC.

The visual structural performance of this example 2mm isotropic multi-TI strategy, utilising three sequences of TI 400, 500 & 600ms, is shown in **online supplement table S9**. Performance is promising and may well improve with further enhancement of resolution.

		**T1w PLIC Specific Sequence (Generation 4 – TI 400ms)**<br><br>**n= 22 infants (%)**	**T1w Neonatal Optimised Multi-TI Imaging Strategy**<br><br>**n= 11 infants (%)**
**Structures Visualised**	**Optic Nerves**	3/22 (14)	10/11 (91)
	**Inner Ear Structures**	0/22 (0)	0/11 (0)
	**Vestibulocochlear Nerve**	0/22 (0)	0/11 (0)
	**Pituitary**	4/22 (18)	6/11 (55)
	**Choroid plexi**	1/22 (5)	8/11 (73)
	**Ventricles**	0/22 (0)	11/11 (100)
	**Corpus Callosum**	5/22 (23)	6/11 (55)
**Tissues Visualised**	**PLIC Myelin Signal**	19/22 (86)	10/11 (91)
	**Cortex**	14/22 (64)	10/11 (91)
	**Deep Grey Matter**	9/22 (41)	10/11 (91)
	**White Matter**	0/22 (0)	10/11 (91)
	**Gyri**	3/22 (14)	4/11 (36)

Online Supplement Table S9. 64mT visual tissue/structural performance of T1w PLIC Specific 400ms Sequence & T1w multi-TI strategy. For the 400ms sequence, averaging has been used when repeat acquisitions are available (5 infants) – median number of acquisitions = 1 (Interquartile Range 1-1). Multi-TI T1w imaging strategy consists of 3x 2mm isotropic acquisitions of TI 400, 500 & 600ms – total scan time =20 min 45 seconds. If serial scans available for any individual infant, the first scan is used for analysis. Posterior Limb of the Internal Capsule (PLIC), Inversion Time (TI).

Congruous and Non-Identified Pathologies

Congruous and non-identified pathologies on 64mT imaging, when compared with 3T reference imaging, are listed for T2w sequence generations in **online supplement table S10**, and for T1w sequence generations in **online supplement table S11**. Examples of non-identified pathologies on 64mT imaging are provided in **online supplement figure S12** - white matter punctate lesions, and **online supplement figure S13** – small cystic lesions.

**Sequence Generations**	**Congruous and non-identified pathology: 64mT Vs 3T T2w imaging**	
	**Congruous Pathology**	**Non-identified Pathology**
**0-4**<br><br>**n= 10 infants**	• Extracranial soft tissue mass<br>• Thalamic atrophy<br>• Occipital lobe cyst<br>• Increased extra-axial CSF space	• Focal parenchymal deep grey matter and white matter punctate lesions<br>• Extra-axial haemorrhage<br>• High T2 signal in PLIC (preterm)<br>• Established cortical infarction<br>• Enlarged cisterna magna<br>• Posterior globe colobomas
**5-7**<br><br>**n= 34 infants**	• Cortical infarction<br>• Thalamic atrophy<br>• Occipital lobe cyst<br>• Increased extra-axial CSF space<br>• Intra-ventricular Haemorrhage<br>• Ventricular asymmetry<br>• Dilated ventricles<br>• Enlarged cysterna magna<br>• Subependymal cysts<br>• White matter and cortical infarction<br>• Abnormal white matter T2 signal<br>• Cystic infarction<br>• White matter volume loss<br>• Hypoplastic cerebellar vermis<br>• Hypoplastic midbrain / brainstem<br>• Polymicrogyria<br>• Agenesis/hypoplasia of corpus callosum<br>• Radial orientation of gyri<br>• Colpocephaly<br>• Extra-axial haemorrhage<br>• Haemorrhagic infarction<br>• Infarction in Posterior Cerebral Artery territory, with secondary compression of the right occipital horn of the lateral ventricle due to tissue swelling<br>• Hypoxic-Ischaemic injury to: white matter, thalamus, basal ganglia,	• Small cystic degeneration within the parietal white matter<br>• Small white matter punctate lesions<br>• Cerebellar microhaemorrhages<br>• Old cerebellar Haemorrhage<br>• Periventricular pseudocysts<br>• Reduced cortical folding for age<br>• Slightly dysplastic cerebellar foliation<br>• Shortened corpus callosum<br>• Small cystic degeneration in basal ganglia<br>• Discreet focal white matter infarction (however, apparent on 64mT PLIC-Specific low resolution T1w images)<br>• Abnormal white matter striations consistent with abnormal migrated cells<br>• Abnormal signal intensity focus in left thalamus, punctate abnormal signal in right lentiform nucleus<br>• (On diffusion only: infarct of right caudate head, abnormal signal ALIC, posterior central sulcus, anterior corpus callosum)

	hippocampi, mesencephalon and brain stem<br>• Subcortical Highlighting<br>• Enlarged cisterna magna<br>• Molar Tooth sign / Batwing fourth ventricle	

Online Supplement Table S10. Congruous & non-identified pathology on 64mT T2w imaging when compared with 3T reference standard T2 weighted structural sequences. More than one sequence generation may be performed on an individual infant. Cerebrospinal Fluid (CSF)

Sequence Generation	Congruous and non-identified pathology: 64mT Vs 3T T1w imaging	
	**Congruous Pathology**	**Non-identified Pathology**
0-1<br>n= 7 infants	• Extra-axial haemorrhage<br>• IVH	• White matter punctate lesions<br>• Increased T1 optic radiations<br>• Cerebellar haemorrhage<br>• Left thalamic hyperintense T1 lesions<br>• Germinolytic cysts<br>• Subdural haemorrhage<br>• Cortical infarction<br>• Enlarged cisterna magna<br>• High T1 signal along left transverse sinus (thrombosis on MRV)<br>• Small frontal lobes<br>• Dilated ventricles<br>• Polymicrogyria<br>• Increased T1 signal dentate nucleus
2<br>n= 17 infants	• IVH<br>• Enlarged cisterna magna<br>• Subdural haemorrhage<br>• Extra-axial haemorrhage<br>• Cephalhaematoma<br>• Deformity of scalp fat suggestive of skull fracture (confirmed on CT)<br>• Small frontal lobes<br>• Dilated lateral ventricles<br>• Agenesis of corpus callosum<br>• Colpocephaly<br>• Cortical, white matter and DGM haemorrhagic infarctions<br>• Widened extra-axial CSF space / interhemispheric fissure<br>• Patchy low T1 signal in white matter<br>• Abnormal Signal - BGT, brainstem, marked cortical highlighting	• Cortical infarction<br>• High T1 signal along left transverse sinus (thrombosis on MRV)<br>• Cerebellar microhaemorrhages<br>• Punctate white matter lesions<br>• Polymicrogyria<br>• Increased T1 signal in dentate nucleus<br>• Radiating pattern of medial gyri<br>• Small cystic degeneration in basal ganglia<br>• Small white matter cysts<br>• Reduced cortical folding for age<br>• (On diffusion only: infarct of right caudate head, abnormal signal ALIC, posterior central sulcus, anterior corpus callosum)

	• Focal infarctions of cortex and white matter	
3<br><br>n= 6 infants	• Severely hypoplastic cerebellar vermis<br>• hypoplastic mid brain<br>• Molar Tooth sign<br>• Increased extra-axial spaces<br>• Prominent lateral ventricles	• Cerebellar microhaemorrhages<br>• Punctate white matter lesions<br>• Small periventricular white matter cysts<br>• Subependymal cysts
4 & Low Resolution T1w imaging<br><br>n= 61 infants	• Abnormal high T1w signal secondary to HIE in the DGM, mesencephalon, brainstem, multiple T1w focal cortical highlighting<br>• Subdural haemorrhages<br>• Interhemispheric dilatation,<br>• Hypoplastic cerebellar hemispheres and vermis<br>• Dilated lateral ventricles<br>• Dilated quadrigeminal cistern<br>• Enlarged cisterna magna<br>• Wide thalamic adhesion<br>• Reduced volume white matter<br>• Abnormal T1w signal – deep grey matter, thalamus, and brainstem<br>• Cortical Highlighting<br>• Widened extra-axial CSF space<br>• Agenesis of corpus callosum<br>• Colpocephaly<br>• Intraventricular haemorrhage<br>• Extracerebral haemorrhage<br>• Abnormal T1w cortical signal<br>• Patchy low T1 signal in white matter<br>• Haemorrhagic infarction<br>• Infarction of cortex & white matter<br>• Focal thalamic high signal intensities<br>• White matter punctate lesions<br>• Small periventricular cyst<br>• Abnormal hippocampal formations<br>• Reduced cortical folding for age<br>• Turricephaly / dolichocephaly / Frontal Bossing	• Low signal T1 in white matter (DEHSI-related)<br>• Caudothalamic cysts<br>• Subependymal cysts<br>• Thin corpus callosum<br>• Small residual IVH<br>• Mild dilatation of lateral ventricles<br>• Short corpus callosum / hypoplastic corpus callosum<br>• Absent splenium<br>• Partial absence of the septum pellucidum<br>• Punctate white matter lesions / microhaemorrhages<br>• Widening of the interhemispheric fissure<br>• Polymicrogyria / abnormal cortical folding<br>• Abnormal white matter striations consistent with abnormal migrated cells<br>• Linear abnormalities on thalami<br>• Posterior globe colobomas<br>• Vestibular dysplasia<br>• Hypoplastic cerebellar vermis & midbrain<br>• Molar Tooth sign<br>• Dysplastic cerebellar foliation<br>• Abnormal hippocampal formations<br>• Small periventricular cyst<br>• Old cerebellar haemorrhages<br>• Punctate abnormal signal in lentiform nucleus<br>• (On diffusion: bilateral focal restriction in the hippocampi, abnormal signal intensity focus in left thalamus, punctate abnormal signal in right lentiform nucleus / On diffusion: infarct of right caudate head, abnormal signal ALIC, posterior central sulcus, anterior corpus callosum)

Online Supplement Table S11. Congruous and non-identified pathology: 64mT Vs 3T T1w structural imaging. More than one sequence generation may be performed on an individual infant. Computerised Tomography (CT), Magnetic Resonance Venography (MRV), Deep Grey Matter (DGM), Basal Ganglia Thalami (BGT), Hypoxic Ischaemic Encephalopathy (HIE), Diffuse Excessive High Signal Intensity (DEHSI), Intra-Ventricular Haemorrhage (IVH).

Non-Identified Pathology – Punctate White Matter Lesions, Microcysts & Subependymal Cyst

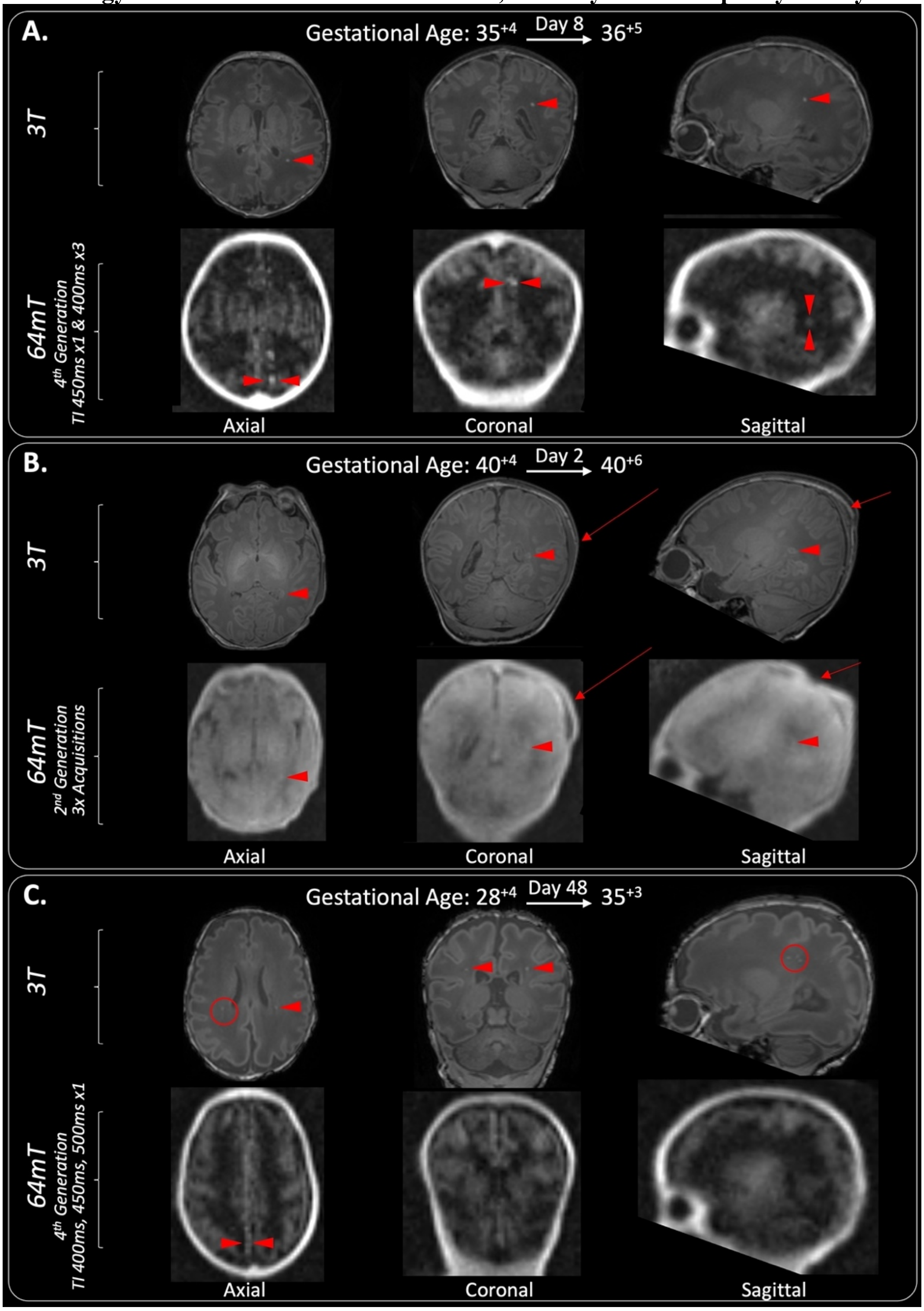

Online Supplement Figure S12. Case series of infants with white matter punctate lesions on T1 weighted imaging at 3T. Single punctate lesions are demarcated with single narrow arrowhead, and punctate clusters are demarcated with a circle. Punctate lesions are commonly identified on preterm infant's MR imaging (A & C). Current T1w sequences at 64mT do not adequately identify punctate lesions, though zipper artefact (double arrowheads) may be misinterpreted as punctate lesions. Within case B, a punctate lesion is identified, in a term infant, ipsilateral to a site of a skull fracture (confirmed on CT, suggested by deformity of soft tissues on MR imaging - arrow) – a corresponding indistinct focus of T1 signal is identified on the 64mT series, but requires corroboration from 3T images to confirm this focus as a white matter punctate lesion.

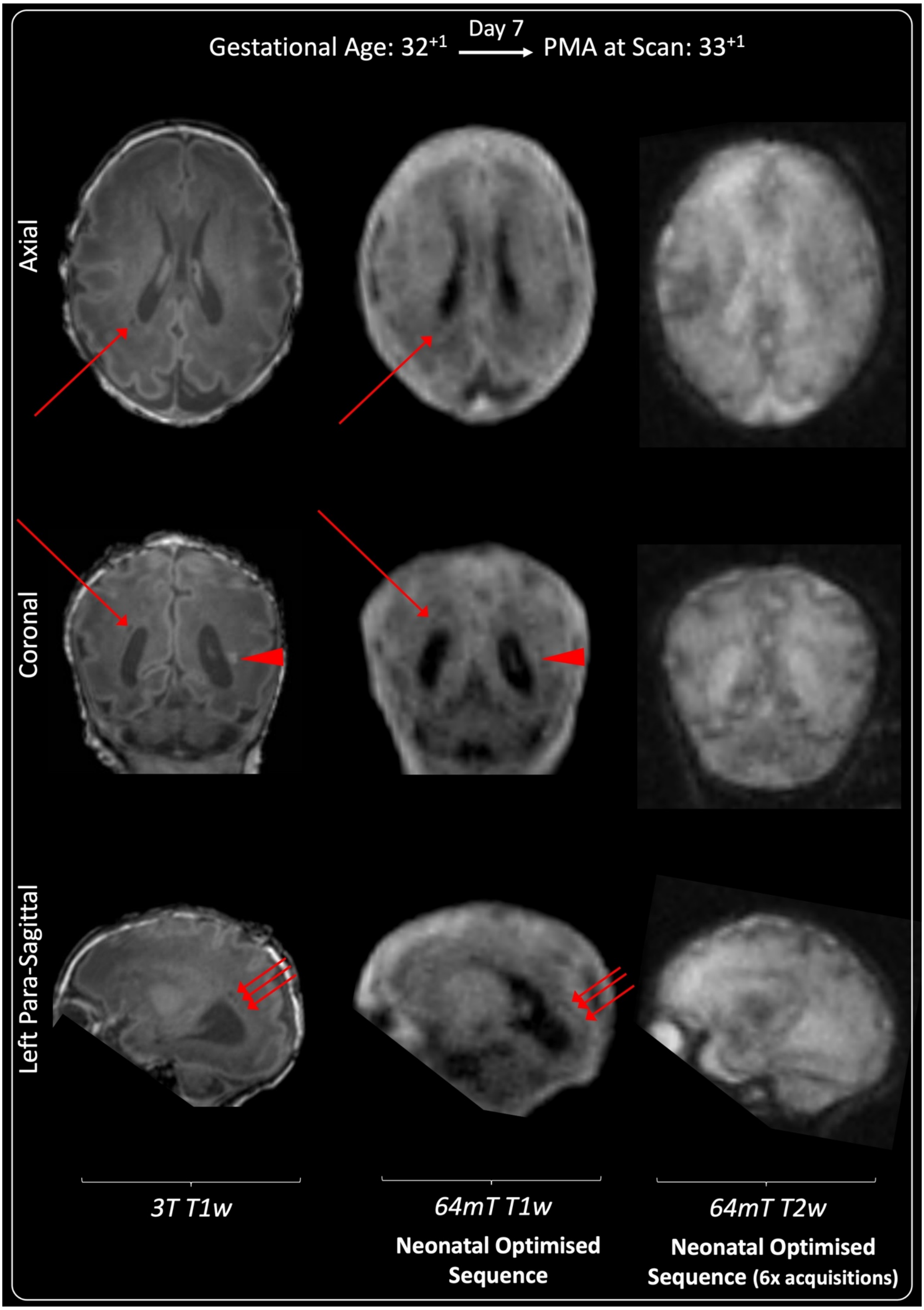

Online Supplement Figure S13. Preterm infant – imaged day 7 on 64m, day 6 on 3T. Ill-defined multiple microcystic lesions visible in bilateral periventricular distribution on 3T MPRAGE imaging (red arrows). Punctate white matter lesion also shown (arrowhead). Some lesions may have putative correlated findings on 64mT imaging – though these areas on ULF slices are indistinct from other areas of noise. 3T T2w imaging was not performed due to infant waking.

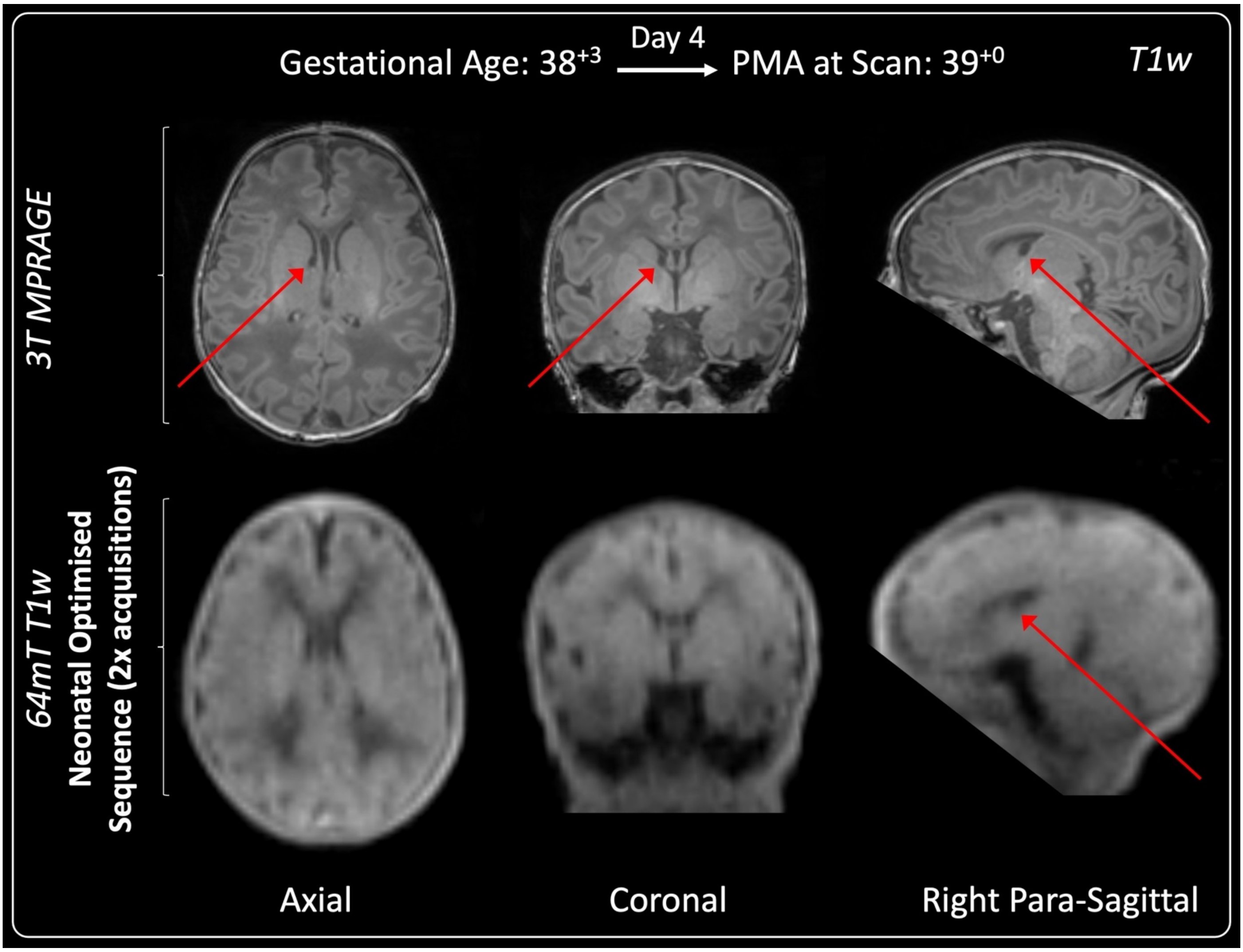

Online Supplement Figure S14. T1w imaging for a term infant with a right-sided subependymal cyst. This is well identified on 3T MPRAGE, but indistinct on 64mT T1w sagittal views and not identified on 64mT T1w axial and coronal views.

Example Imaging: Motion Artefact

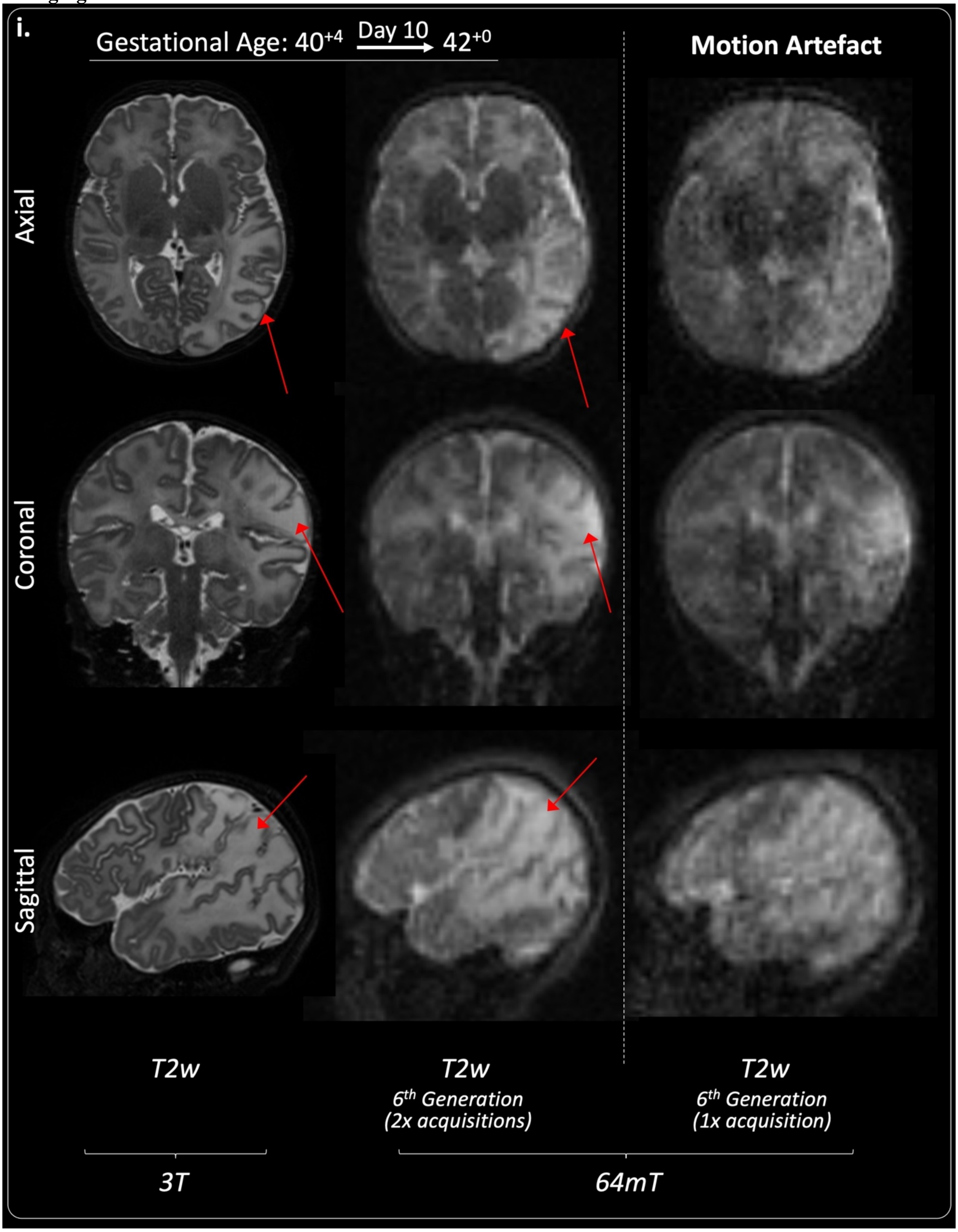

Online Supplement Figure S15. Diffuse left sided white matter and cortical parieto-occipital infarction. Infants with brain injury will be prone to motion during imaging. As is the case in this series, one of the T2w acquisitions were severely distorted by motion artefact (Right hand column), at 64mT motion artefact may be occult and present as blurring.

Example Imaging: Pituitary & Pituitary Stalk

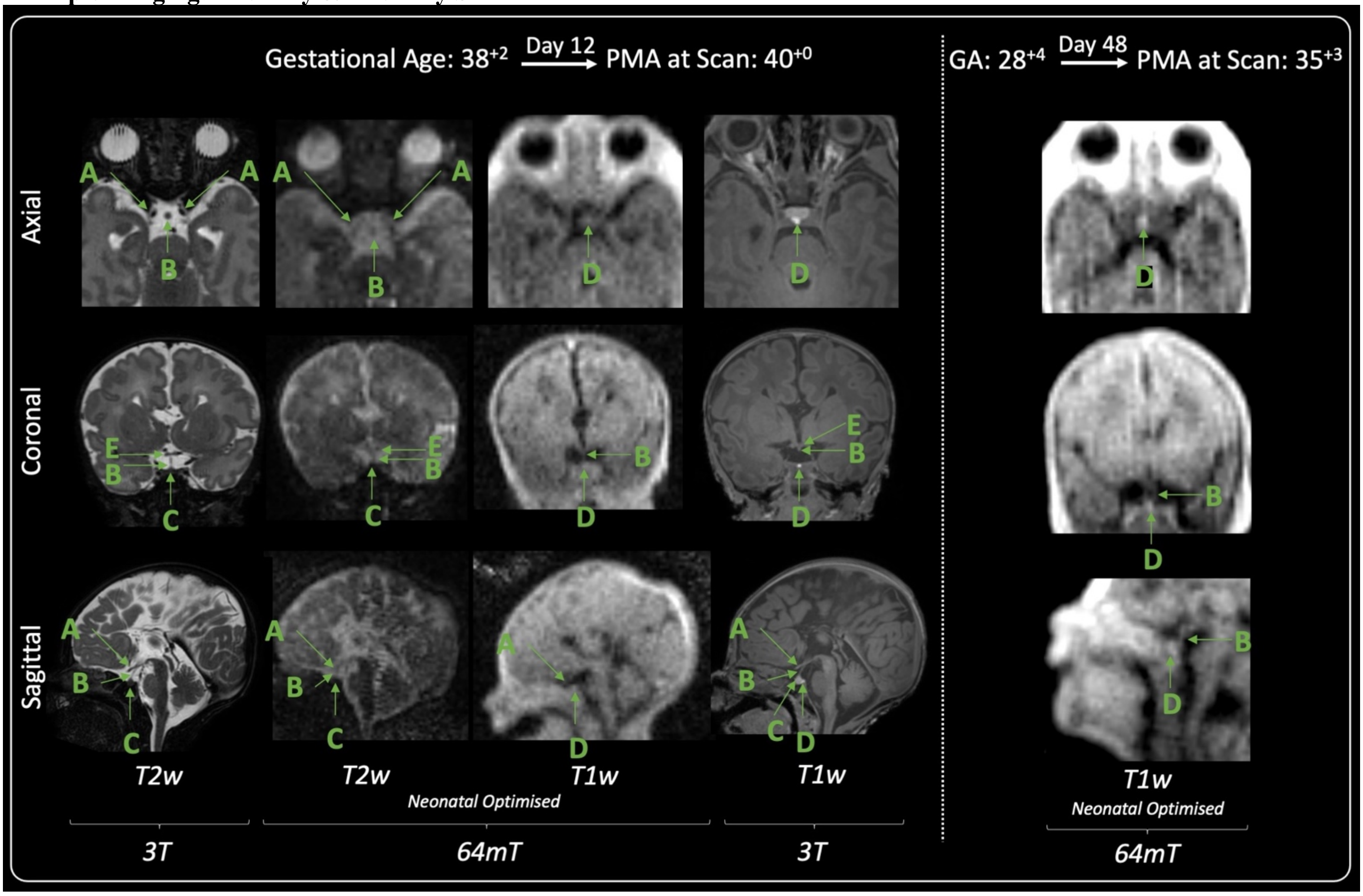

Online Supplement Figure S16. T1w and T2w axial, coronal and sagittal slices are show at the level of the pituitary. *Infant on left-hand side has congenital absence of the corpus callosum: A) Optic nerves, B) Pituitary stalk, C) Pituitary Gland, D) (Putative) Posterior Pituitary Gland, E) Optic Chiasm.*

Example Imaging: Developmental Brain Abnormalities

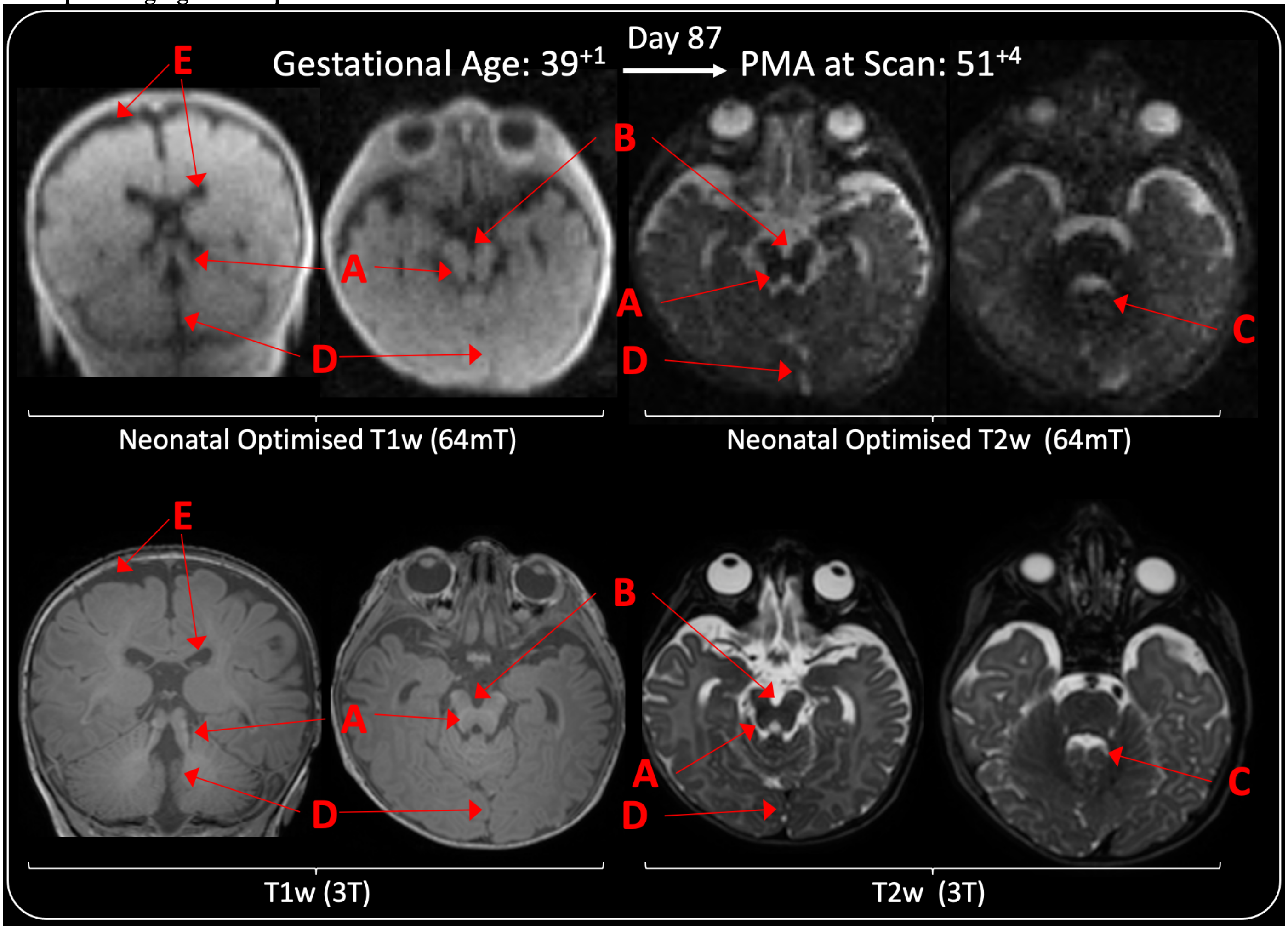

Online Supplement Figure S17. Congenital Structural Anomalies. *Thickened cerebellar peduncles (A) and deep interpeduncular fossa (B) leading to the characteristic 'Molar Tooth Sign' of Joubert's (A & B)* and a 'Batwing' appearance of the fourth ventricle (C), the two cerebellar hemispheres are in apposition in midline (D) due to severe vermian hypoplasia. There is mild increase in the extra-axial and ventricular CSF spaces (E). PMA: Post-Menstrual Age. *Nag C, Ghosh M, Das K, Ghosh T. Joubert syndrome: The molar tooth sign of the mid-brain. Ann Med Health Sci Res 2013;* ***3****: 291.*

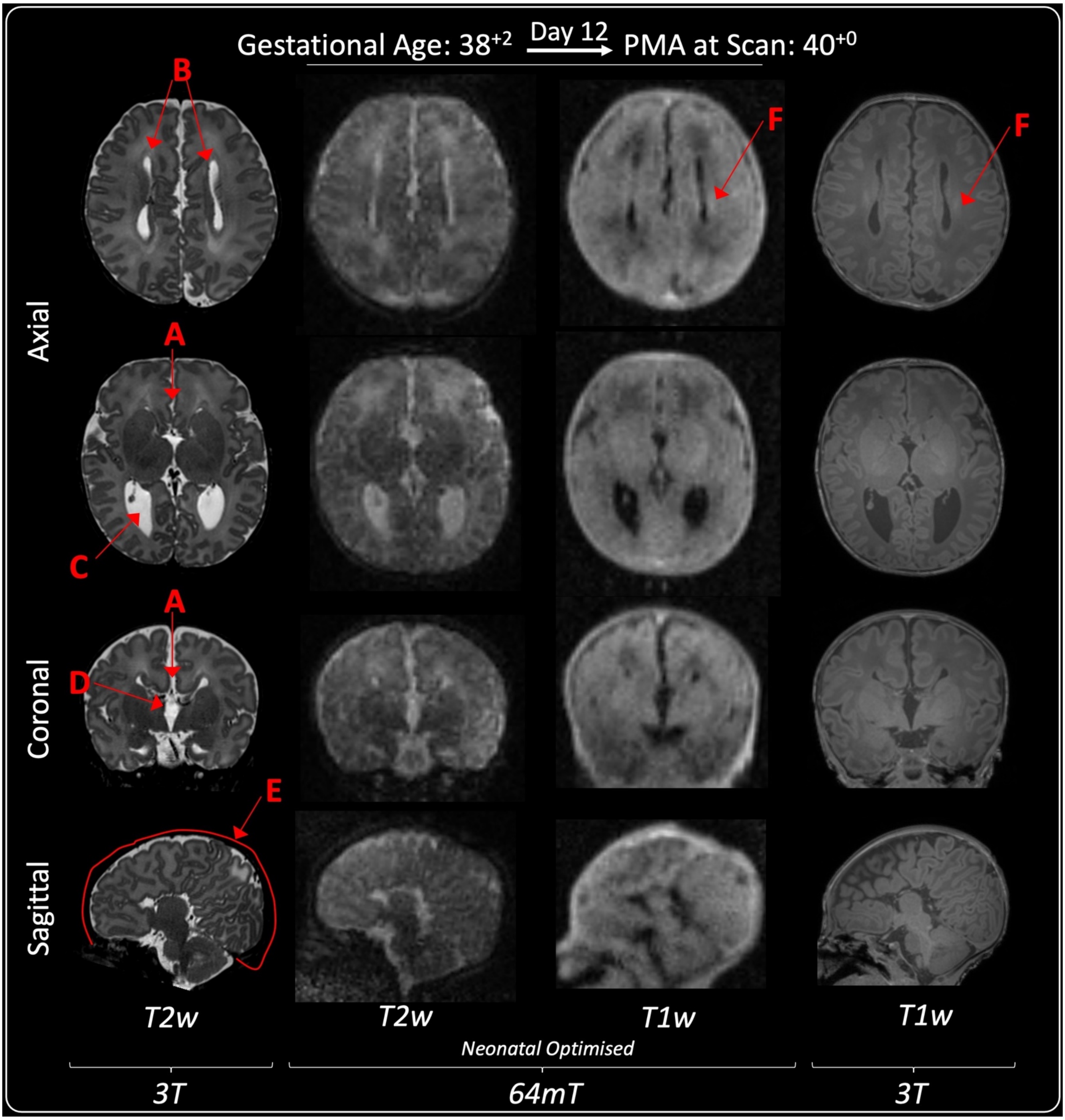

Online Supplement Figure S18. Congenital Structural Anomalies. *A) Agenesis of the corpus callosum, B) parallel orientation of lateral ventricles, C) colpocephaly, D) high riding third ventricle, E) radiating pattern of the medial sulci. F) T1 signal, consistent with myelination within the superior cortical spinal tract. GA: Gestational Age, PMA: Post-Menstrual Age.*

UNITY Consortium Members

First name / Initial	Surname	Relevant affiliation
Amanda	Adu-Amankwah	Korle-Bu Teaching Hospital, Accra, Ghana
Kenneth	Ae-Ngibise	Kintampo Health Research Centre, Kintampo, Ghana
Tomoki	Arichi	St Thomas Hospital, King's College London, UK
Sharada	Balaji	University of British Columbia, Vancouver, Canada
Levente	Baljer	Centre for Neuroimaging Sciences, King's College London, UK
Arpan	Banerjee	National Brain Research Centre India
Peter	Basser	National Institute of Health, USA
Jennifer	Beauchemin	Advanced Baby Imaging Lab, Rhode Island Hospital, Providence, USA
Carly	Bennallick	Centre for Neuroimaging Sciences, King's College London, UK
Yemane	Berhane	Addis Continental Institute Of Public Health, Addis Ababa, Ethiopia
Niall	Bourke	Centre for Neuroimaging Sciences, King's College London, UK
Layla	Bradford	CUBIC, University of Cape Town, South Africa
Phoebe	Burton	Advanced Baby Imaging Lab, Rhode Island Hospital, Providence, USA
Paul	Cawley	St Thomas Hospital, King's College London, UK
Mara	Cercignani	CUBRIC, Cardiff University, UK
Karen	Chetcuti	Blantyre Malarial Project, University of Malawi College of Medicine, Malawi
Cowles A	Chilingulo	Blantyre Malarial Project, University of Malawi College of Medicine, Malawi
Alexica	De canha	University of Pretoria, South Africa
Doug	Dean III	University of Wisconsin-Madison, USA
Jaclyn	Delarosa	Path, Seattle, USA
Sean C	Deoni	Maternal, Newborn, and Child Health Discovery & Tools, Bill & Melinda Gates Foundation; Seattle USA
Kirsten	Donald	CUBIC, University of Cape Town, South Africa
Adam	Dvorak	University of British Columbia, Vancouver, Canada
David	Edwards	St Thomas Hospital, King's College London, UK
Heidi	Frail	Hyperfine, Inc. Guilford, Connecticut, USA
Joseph	Hajnal	St Thomas Hospital, King's College London, UK
William	Hollander	CaliberMRI, Boulder, Colorado, USA
Zahra	Hoodbhoy	Aga Khan University Hospital, Karachi, Pakistan
Fyezaah	Jehan	Aga Khan University Hospital, Karachi, Pakistan
Derek	Jones	CUBRIC, Cardiff University, UK
Margaret	Kasaro	UNC Department of Obstetrics and Gynecology, Lusaka, Zambia
Able	Khosa	Training & Research Unit of Excellence, Zomba, Malawi
Shannon	Kolind	University of British Columbia, Vancouver, Canada
Samson	Lecurieux Lafayette	St Thomas Hospital, King's College London, UK
Anne CC	Lee	Harvard Medical School, Boston, MA, USA
Beatrice	Lena	Leiden University Medical Centre, Netherlands

Emil	Ljungberg	Lund University, Sweden
Yaw	Mensah	Korle-Bu Teaching Hospital, Accra, Ghana
Rosalyn	Moran	Centre for Neuroimaging Sciences, King's College London, UK
Joan	Murungi	Makerere University, Kampala, Uganda
Victoria	Nankabirwa	Makerere University, Kampala, Uganda
Blessings	Nthulula	Training & Research Unit of Excellence, Zomba, Malawi
Solomon	Nyame	Kintampo Health Research Centre, Kintampo, Ghana
Samuel	Oppong	Korle-Bu Teaching Hospital, Accra, Ghana
Francesco	Padormo	Hyperfine, Inc. Guilford, Connecticut, USA
Sant-Rayn	Pasricha	Walter and Eliza Hall Institute of Medical Research, Parkville, Australia
Michael	Pepper	University of Pretoria, South Africa
Kwaku	Poku Asante	Kintampo Health Research Centre, Kintampo, Ghana
Jessica	Ringshaw	CUBIC, University of Cape Town, South Africa
Hemmen	Sabir	University Hospital at Bonn, Germany
Lydia	Sekoli	University of Pretoria, South Africa
Jeffrey SA	Stringer	UNC Department of Obstetrics and Gynecology, Lusaka, Zambia
Terrie	Taylor	Blantyre Malarial Project, University of Malawi College of Medicine, Malawi
Rui Pedro A. G.	Teixeira	Hyperfine, Inc. Guilford, Connecticut, USA
Pip	Torelli	Hyperfine, Inc. Guilford, Connecticut, USA
Chip	Truwit	Hyperfine, Inc. Guilford, Connecticut, USA
Methodius	Tuuli	Korle-Bu Teaching Hospital, Accra, Ghana
Jeanne	Van Rensburg	University of Pretoria, South Africa
Frantisek	Vasa	Centre for Neuroimaging Sciences, King's College London, UK
Maclean	Vokhiwa	Training & Research Unit of Excellence, Zomba, Malawi
Andrew	Webb	Leiden University Medical Centre, Netherlands
Callie	Weiant	CaliberMRI, Boulder, Colorado, USA
Neale	Wiley	University of British Columbia, Vancouver, Canada
Steve	Williams	Centre for Neuroimaging Sciences, King's College London, UK

Online Supplement References

1. GIRFT. Neonatology Review - Unit Report. Getting It Right First Time 2020.
2. ONS. UK Census: Lambeth & Southwark. 2021. [Internet – Last accessed: 2023 August 19]. Available from: https://www.ons.gov.uk/visualisations/censuspopulationchange/
3. Padormo F, Cawley P, Dillon L, et al. In vivo T1 mapping of neonatal brain tissue at 64 mT. *Magn Reson Med* 2023; **89**: 1016–25
