# Supplementary material for: Development of neonatal-specific sequences for portable ultralow field magnetic resonance brain imaging: a prospective, single-centre, cohort study
Source: eClinicalMedicine. 2023 Oct 10;65:102253. doi: 10.1016/j.eclinm.2023.102253 (PMC10725077; doi:10.1016/j.eclinm.2023.102253)
Supplement: UNITY Consortium—PubMed Study Group Indexing [file mmc2.docx]

| **UNITY Consortium – PubMed Study Group Indexing** | |
| --- | --- |
| **First name / Initial** | **Surname** |
| Sean C. | Deoni |
| Emil | Ljungberg |
| Carly | Bennallick |
| Shannon | Kolind |
| Doug | Dean III |
| Michael S. | Pepper |
| Lydia | Sekoli |
| Alexica | De Canha |
| Jeanne | Van Rensburg |
| Derek K. | Jones |
| Niall | Bourke |
| Hemmen | Sabir |
| Samson | Lecurieux Lafayette |
